# A splice donor variant in *SLAMF1* is associated with canine atopic dermatitis

**DOI:** 10.3389/fvets.2025.1550617

**Published:** 2025-06-19

**Authors:** Oliver P. Forman, Jamie Freyer, Abigail Kerr, Julia D. Labadie, Michael Denyer, Debbie J. Gow, Janet Alexander, Michelle Daya, Yaindrys Rodriguez Olivera, Cecilia Lozoya, Christian Leutenegger, Christian Savard, Jason T. Huff, Rebecca Chodroff Foran

**Affiliations:** ^1^Wisdom Panel, Science and Diagnostics, Mars Petcare, Los Angeles, CA, United States; ^2^Veterinary Specialists Scotland, Linnaeus Veterinary Limited, Mars Veterinary Health, Livingston, United Kingdom; ^3^Waltham Petcare Science Institute, Mars Petcare, Melton Mowbray, United Kingdom; ^4^Biovet (A Division of Antech Diagnostics Inc.), Science and Diagnostics, Mars Petcare, Saint-Hyacinthe, QC, Canada; ^5^Antech Diagnostics Inc., Science and Diagnostics, Mars Petcare, Fountain Valley, CA, United States

**Keywords:** CAD, atopy, dermatitis, canine, allergy, SLAMF1, atopic dermatitis, allergic dermatitis

## Abstract

**Introduction:**

Canine atopic dermatitis (CAD) is a common inflammatory skin condition in dogs. It is a lifelong issue that poses a significant welfare concern due to the chronic skin discomfort and pruritus (itching) experienced by affected animals. Excessive scratching, licking, and chewing cause self-inflicted injuries to the skin and increase the risk of secondary infections. Several dog breeds, including Labrador Retriever, Boxer, and French Bulldog, are known to be predisposed to these issues, suggesting a genetic link to the condition.

**Methods:**

Access to a large population of dogs genotyped on a medium-density single-nucleotide polymorphism (SNP) array through commercial Wisdom Panel testing, along with their linked clinical records, allowed a large-scale, highly powered genome-wide association study (GWAS) to be performed. In this study, over 28,000 dogs were examined to identify genetic changes associated with CAD.

**Results:**

A statistically significant signal on canine chromosome 38 was identified, with a particularly strong signal in French Bulldogs. Whole-genome resequencing revealed a compelling splice donor variant in the signaling lymphocytic activation molecule 1 (*SLAMF1*), a transmembrane receptor with important functions in immune cells. Further analysis of additional genome sequences and RNA samples from the MARS PETCARE BIOBANK confirmed that the *SLAMF1* splice variant is a strong potential contributor to an increased risk of atopic dermatitis.

**Discussion:**

The discovery represents the first compelling genetic variant associated with CAD to be validated in more than one breed of dog. The study identifies *SLAMF1* as a potential pharmaceutical target and the associated variant as a biomarker to enable dog breeders to make informed breeding decisions to reduce risk of CAD in future generations. The presence of the *SLAMF1* variant in many dog breeds and free-roaming dogs worldwide, indicates its potential role in contributing to the global risk of CAD.

## Introduction

Canine atopic dermatitis (CAD) is a lifelong inflammatory skin condition that is commonly encountered in veterinary practices. Its overall prevalence varies by population; however, it is estimated to affect up to 30% of dogs (1, 2). Despite its prevalence, the pathogenesis of CAD is complex and not fully understood. Our current understanding is that a combination of heritable and environmental factors contributes to immune dysregulation, including the increased production of immunoglobulin E (IgE), skin barrier defects, and alterations in the cutaneous microbiome, thereby allowing for the development of an allergic phenotype (3).

Initial clinical signs include inflammation (erythema) of the skin and pruritus, which may present as scratching, rubbing, chewing, licking, or head shaking. Over time, these can lead to self-inflicted injuries of the skin, alopecia, crusting, hyperpigmentation, thickening of the skin (lichenification), and secondary bacterial and yeast infections (4). Irritation from CAD commonly occurs in the paws, pinnae, face, axillae, and groin regions (5). The diagnosis of CAD is challenging because there are no specific diagnostic tests available. Therefore, diagnosis depends on a detailed medical history, physical examination findings, and elimination of other causes of pruritus, such as ectoparasites, skin infections, and food or flea allergies (6). Management of CAD typically involves anti-inflammatory medications, dietary adjustments, and avoidance of allergens; however, there is currently no definitive cure (7).

A deeper understanding of CAD pathogenesis would enable the development of new diagnostic and management strategies for this chronic condition. The pronounced breed-specific predisposition to CAD observed in breeds such as the Golden Retriever, Labrador Retriever, Boxer, West Highland White Terrier (WHWT), French Bulldog, and German Shepherd suggests that genetics plays a key role in the development of the disease (5). CAD heritability has been estimated at 0.47 in Labrador and Golden Retrievers and 0.31 in WHWTs (8, 9).

In addition, several genome-wide association studies (GWASs) focused on CAD have been conducted, and numerous single-nucleotide polymorphisms (SNPs) have been identified (10). However, many of these studies are limited in power due to small cohort sizes and low genotyping density. An early GWAS of Golden Retrievers that utilized a low-density 22 k SNP microarray identified two significant intergenic SNPs on chromosomes 2 and 3. (11). Two separate cohorts of atopic Labrador Retrievers and WHWTs showed an association with elevated dust-mite-specific IgE on chromosomes 5 and 35, respectively (12, 13). In a Swedish cohort of German Shepherd dogs, a GWAS involving 91 cases and 88 controls identified a significant association on chromosome 27 in the region encoding Plakophilin 2, which is an important protein for skin structure. Although this finding seemed promising, further analysis revealed no difference in the expression of this gene between CAD cases and control groups (14). Conflicting observations have also been reported regarding WHWTs from different geographical areas. An Australian dog population showed a CAD association with a 1.3 Mb region on chromosome 17 (15), while an American population showed a CAD association with a 2.7 Mb region on chromosome 3 (16). Finally, a recent GWAS identified a variant in the interleukin 4 receptor that reduces the risk of CAD in miniature Dachshunds, possibly by impairing the receptor and reducing the downstream inflammatory pathways associated with CAD (17). Previous studies have demonstrated the complexity and genetic heterogeneity of CAD both across and within breeds, highlighting the need for large cohorts to sufficiently power studies. The introduction of genetic testing using a commercial 100 k SNP genotyping array in veterinary clinics, starting at an early age and as part of large-scale longitudinal biobanking studies, provides a unique opportunity to accumulate large numbers of diagnosed cases of multiple disorders over time. Using a cohort accumulated over a 5-year period and derived from a clinico-genetics dataset of over 1.2 million dogs, we conducted a GWAS to identify genetic associations in dogs diagnosed with atopic dermatitis.

## Methods

### Sampling

DNA samples were collected via commercial testing with Wisdom Panel™ Premium, Wisdom Panel™ Essential, and Optimal Selection™ retail products, along with genetic testing performed as part of Optimum Wellness Plans® for puppies at Banfield Pet Hospital branches (Vancouver, WA, United States) and as part of the MARS PETCARE BIOBANK™ (18). The samples were collected either through non-invasive cheek swabbing by dog owners or veterinary professionals or through blood sampling by a veterinary professional at a Banfield Pet Hospital, in line with regulations governing diagnostic testing. Consent for the use of DNA data in research was obtained through the client’s agreement to the terms and conditions of DNA testing via Wisdom Panel. Analysis and sequencing of cDNA were performed using samples collected from dogs enrolled in the MARS PETCARE BIOBANK. All samples originated from the United States or Mexico.

### Genotyping

DNA was extracted from whole blood and buccal swabs at Neogen Laboratories (Neogen Corporation, Lincoln, NE, United States). Genotyping was performed using a custom 100 k Illumina Infinium XT SNP microarray (Illumina, Inc., San Diego, CA, United States), also at Neogen Laboratories. The microarray was designed and validated for use following the same protocols and principles previously described (19). Microarray genotyping analyses were carried out following the manufacturer’s standard protocols for the Illumina XT platform (Illumina, Inc.). Only samples achieving a genotyping call rate of at least 95% were included in the study.

### Clinical information

For DNA samples submitted directly for genotyping through Banfield Pet Hospital clinics, data from genotyped dogs were directly linked to clinical records stored in the Banfield electronic medical records (EMRs). For DNA samples collected and submitted by general retail consumers of Wisdom Panel products, data from genotyped dogs were linked to Banfield EMRs through the anonymized cross-matching of pet and owner information, in accordance with personally identifiable information (PII) regulations.

### Inclusion criteria

CAD cases and controls were categorized based on the labeling provided by general veterinary practitioners in the Banfield EMRs and criteria set by a board-certified veterinary dermatologist. Given the retrospective nature of the data, this diagnosis reflects atopic dermatitis in the broad sense and may include dogs with food-allergic dermatitis (8).

CAD cases had to be in the age range of 0.3 to 20 years and had to have received at least 3 months of ectoparasite control. CAD cases also required a diagnosis of recurrent (more than one episode) clinical signs of atopic dermatitis, including atopic/allergic dermatitis, otitis, pododermatitis, pruritus, pyoderma, or Malassezia infections. In addition, CAD cases also needed to have received a prescription for recurrent systemic anti-inflammatories, antihistamines, or medicated ear drops.

Controls had to be in the age range of 3 to 20 years and needed not to have been diagnosed with any of the following recurrent symptoms: Acne, alopecia of undetermined origin, dermatitis (of any type), folliculitis, lichenification, Malassezia, otitis (of any type), paronychia, pododermatitis, pruritus, or pyoderma (of any type). Controls also needed not to have been prescribed recurrent systemic or topical anti-inflammatories, antihistamines, or medicated ear drops.

For breed assignment, single-breed dogs were defined as those with greater than or equal to 80% single-breed ancestry, as determined by the Wisdom Panel breed classification algorithm (BCSYS) (20), as previously described (21).

### Genotype analysis

A genome-wide association study analysis was performed using a linear mixed-model approach in the software package GEMMA v0.98.5, including a centered relatedness matrix (22). PLINK v1.90b6.22 64-bit (23) was used to apply the following quality control: Samples with an overall genotyping success rate lower than 95% across all tested SNPs were filtered out, as were variants with a minor allele frequency (MAF) below 1% or a genotyping success rate lower than 95%. Separate QC filtering was performed for each GWAS. The number of markers available after QC can be found in Supplementary file S1. The significance thresholds on the GWAS plots were set to 0.05 and Bonferroni-corrected for the number of markers after QC for each GWAS. Principal component analysis (PCA) plots were generated using PLINK. All reported genome locations are based on the CanFam4 genome build, unless stated otherwise. The mode of inheritance (MOI) was assessed by fitting a generalized linear model with a logit link function using the R programming language, testing for an association between allele dose and case–control status as follows: For the additive model, the dose was coded as 0, 1, or 2, representing the number of copies of the risk allele. For the dominant model, the dose was coded as 0 or 1, with zero representing the non-risk homozygous genotype and 1 representing the heterozygous or risk homozygous genotype. For the recessive model, the dose was coded as 0 or 1, with zero representing the non-risk homozygous genotype and the heterozygous genotype and 1 representing the risk homozygous genotype. A likelihood ratio test was used to identify the best model fit.

### Whole-genome sequencing

Whole genome sequencing was performed using DNA extracted from buccal swab samples collected via commercial DNA testing. Whole-genome sequencing was performed using a standard methodology to achieve a target read depth of 30x on an Illumina NovaSeq at Neogen Inc., Lincoln, Nebraska, United States. The data were analyzed using the Illumina Dragen pipeline (24) and aligned to the CanFam4 genome assembly. Variants were annotated using SNPeff, and statistical analysis was performed using SNPsift (25). Samtools 1.13 was used to filter the variant set to only include those within the specific chromosomal region of interest (26).

### Extended *SLAMF1* genotyping

Extended genotyping for the signaling lymphocytic activation molecule 1 (*SLAMF1*) candidate variant was performed by LGC Service Lab in the United Kingdom using KASP genotyping methodology (27). The primers were as follows: Primer_AlleleT: ATATGAATCTCTTTATTGTCAGACACCTA, Primer_AlleleC: TTATGAATCTCTTTATTGTCAGACACCTG, and Primer_Common: GAAGTGGTATTACTGCTGTTGAGAAGAA. Cases and controls from the French Bulldog and Boxer breeds, which yielded genome-wide significant results for the *SLAMF1* region, were used for these additional genotyping experiments.

### *SLAMF1* cDNA analysis and sequencing

Expression analysis was performed using RNA extracted from whole blood samples stored in RNAProtect (catalog #76,554; Qiagen, Germantown, MD, United States). RNA was extracted by Qiagen Genomic Services (Frederick, MD, United States), and expression analysis was performed by BioVet Inc. (Antech Diagnostics). Full details of the RNA samples, including concentrations and RIN values, can be found in Supplementary file S2. cDNA synthesis and PCR were completed in a single reaction using the QIAGEN OneStep RT-PCR Kit, with primers TCCCAGCCAACAGTTCTCAC and TAAATGGTGGTGCAGGGGTC, located in exons 3 and 6, respectively.

## Results

### Combined single-breed GWAS

**Figure 1:**
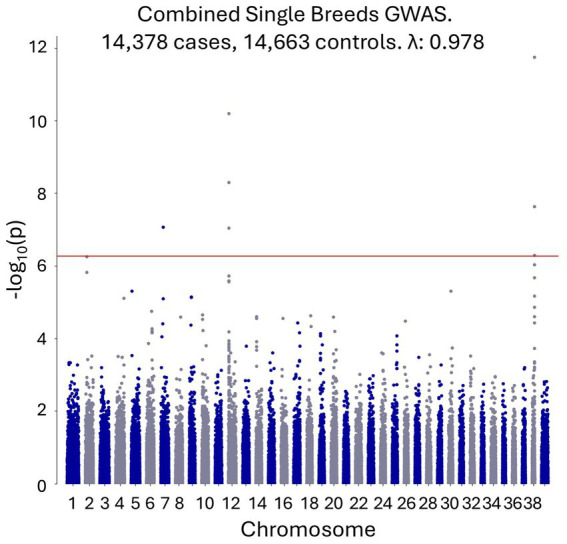
A combined single-breed CAD GWAS shows genome-wide significant signals on canine chromosomes 7, 12 and 38.

An initial GWAS was performed using 14,378 single-breed cases and 14,633 breed-matched controls to tightly control for potential population stratification, with no covariates. The breeds included are shown in Supplementary Table S1. PCA revealed close genetic matching between the cases and controls and clustering of different breeds (Supplementary file S3). Genome-wide significant signals were observed on canine chromosome 7, chromosome 12 (in the dog leukocyte antigen (DLA) region), and chromosome 38, with top SNPs at chr7:26,226,672 (*p* = 8.57×10^−8^), chr12:1,709,085 (*p* = 6.41×10^−11^), and chr38:22,433,504 (*p* = 1.79×10^−12^), respectively (Figure 1). All coordinates refer to the CanFam4 genome build.

### Within-breed GWAS

**Figure 2:**
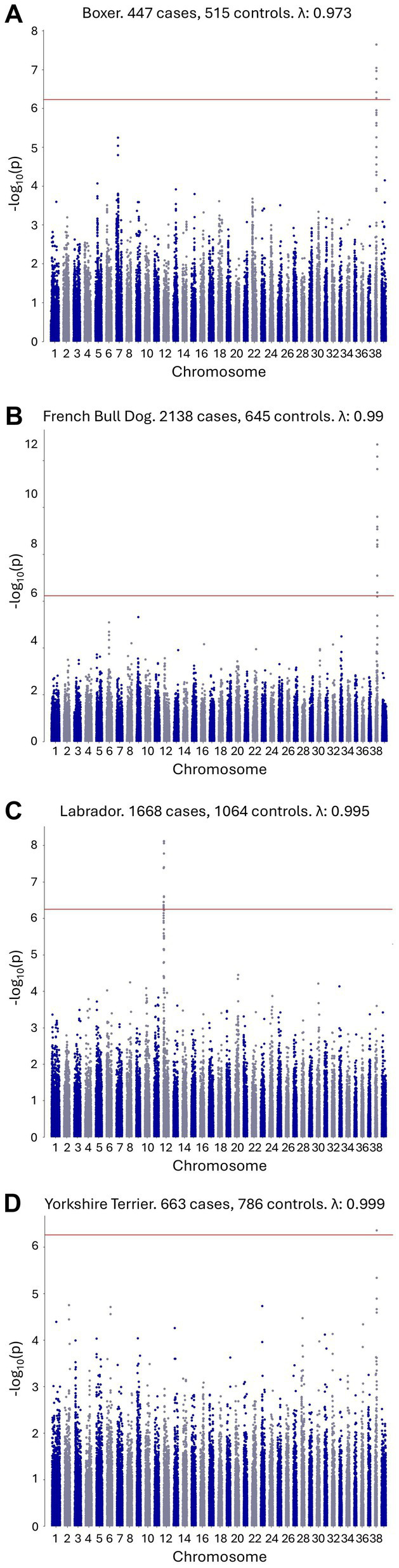
Single-breed CAD GWAS with genome-wide significant signals. **(A)** Boxer CAD GWAS with a genome-wide significant signal on canine chromosome 38. **(B)** French Bulldog CAD GWAS with a genome-wide significant signal on canine chromosome 38. **(C)** Labrador Retriever CAD GWAS with a genome-wide significant signal on canine chromosome 12. **(D)** Yorkshire Terrier CAD GWAS with a genome-wide significant signal on canine chromosome 38.

A within-breed GWAS with no covariates was performed where at least 200 cases and controls were available for a given breed. This included the following breeds, listed from the breed with the highest number of cases to the breed with the lowest number of cases: French Bulldog, Labrador Retriever, German Shepherd dog, Golden Retriever, Shih Tzu, Yorkshire Terrier, Miniature Schnauzer, Boston Terrier, Medium/Standard Poodle, Boxer, Dachshund, Pug, Pembroke Welsh Corgi, Miniature/Toy Poodle, Beagle, Chihuahua, Japanese Shiba Inu, Siberian Husky, and Great Dane. Full case and control numbers are available in Supplementary file S1. Genome-wide significant results were observed for the French Bulldog, Boxer, Labrador Retriever, and Yorkshire Terrier breeds (Figure 2). The top SNP for the French Bulldog, Boxer, and Yorkshire Terrier breeds were at chr38:22,420,361 (*p* = 2.04×10^−13^), chr38:22,263,306 (*p* = 2.27×10^−8^), and chr38:22,433,504 (*p* = 4.38×10^−7^), respectively, representing the same chromosome 38 locus identified in the combined single-breed GWAS. The top SNPs for the Labrador Retriever GWAS was at chr12:1,904,093 (*p* = 9.09×10^−08^) within the DLA region. The chromosome 7 signal identified in the combined single-breed GWAS was not replicated using the single-breed approach. All GWAS results, including QQ plots, are provided in Supplementary file S1.

### Whole-genome sequence analyses

**Figure 3:**
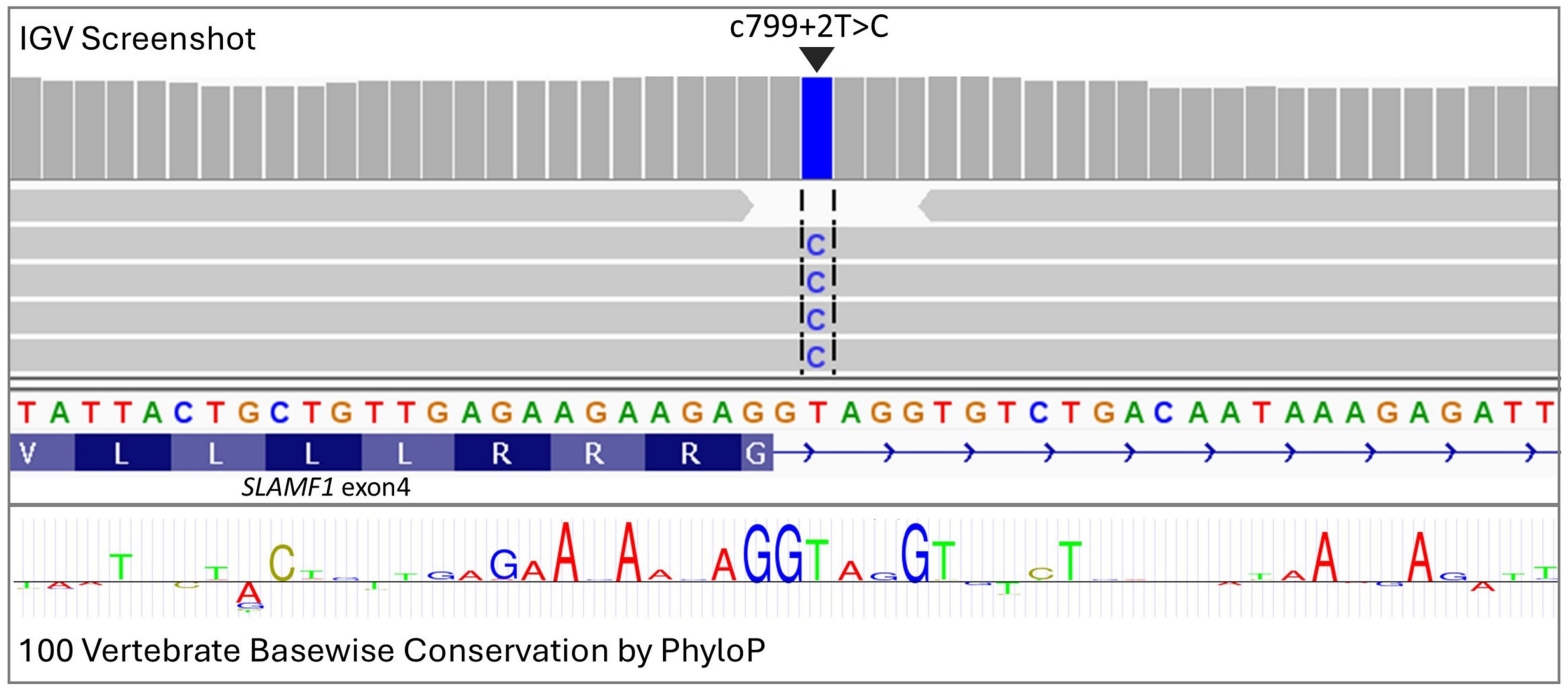
Visualization in IGV of deep sequencing data from an atopic dermatitis case showing a splice donor variant in exon 4 of *SLAMF1* (c799 + 2 T > C). Conservation across 100 vertebrate species is highlighted through the PhyloP track, with letter size being relative to the level of conservation.

A total of 78 genomes were available for analysis, including three case genomes that were sequenced specifically for this study and 75 genomes from earlier studies. The three cases, which consisted of one Boston terrier, one French Bulldog, and one American Pitbull Terrier, were included based on a phenotype consistent with the case definition and homozygosity for disease-associated alleles across the associated interval (chr38:22,129,614-22,543,739). Risk loci disease-associated intervals cannot be precisely defined in the same way as simple recessive loci, which can be mapped using recombination breakpoints. Therefore, the associated interval was defined by empirically extending a region from the top SNPs from the within-breed GWAS. Due to the expected high frequency of the causal variant, an additional seven dogs (four French Bulldogs, one American Staffordshire Terrier, one Boston Terrier, and one Boxer) were defined as haplotypic cases based on the presence of the shared disease-associated homozygous interval (chr38:22,129,614-22,543,739). The genotype data across the region used to define the cases and controls can be found in Supplementary Table S2. Final variant analysis was performed with 10 cases and 68 controls, further extending the disease-associated region empirically around the top SNPs to capture additional variants (chr38: 21805771–22,919,607). Genome sequences were aligned to the CanFam4 genome build, variants were called, and their effects were predicted using SNPeff. Statistical analysis was performed using SNPsift. The variants were ranked based on the *p*-value (SNPsift) and filtered according to consequence prediction (SNPeff). Of the 13,154 unique variants identified, the top-ranking variant, based on both consequence prediction and probability value, was a splice donor variant downstream of *SLAMF1* exon 4 (c799 + 2 T > C, based on NCBI Nucleotide transcript entry: NM_001003084; Figure 3). A full list of the variants identified can be found in Supplementary Table S3. MaxtentScan (28) analysis predicted the splice site to be weakened by the variant (score reduced from 9.65 to 1.90), and GenScan (29) analysis predicted that the splice donor variant may cause exon skipping. No other deleterious or segregating variants were identified (Supplementary Table S3). The region was assessed for structural variants using the Dog10k genome set (30). As part of the Dog10k data release, structural variants were called by the consortium on a high-quality subset of 1,879 genomes using Manta SV (31). No clearly associated structural variants were identified within the associated region. In addition, BAM files from the cases and controls were visually assessed in IGV (32) to check for any structural variants potentially missed by automated calling.

### Gene expression analysis

**Figure 4:**
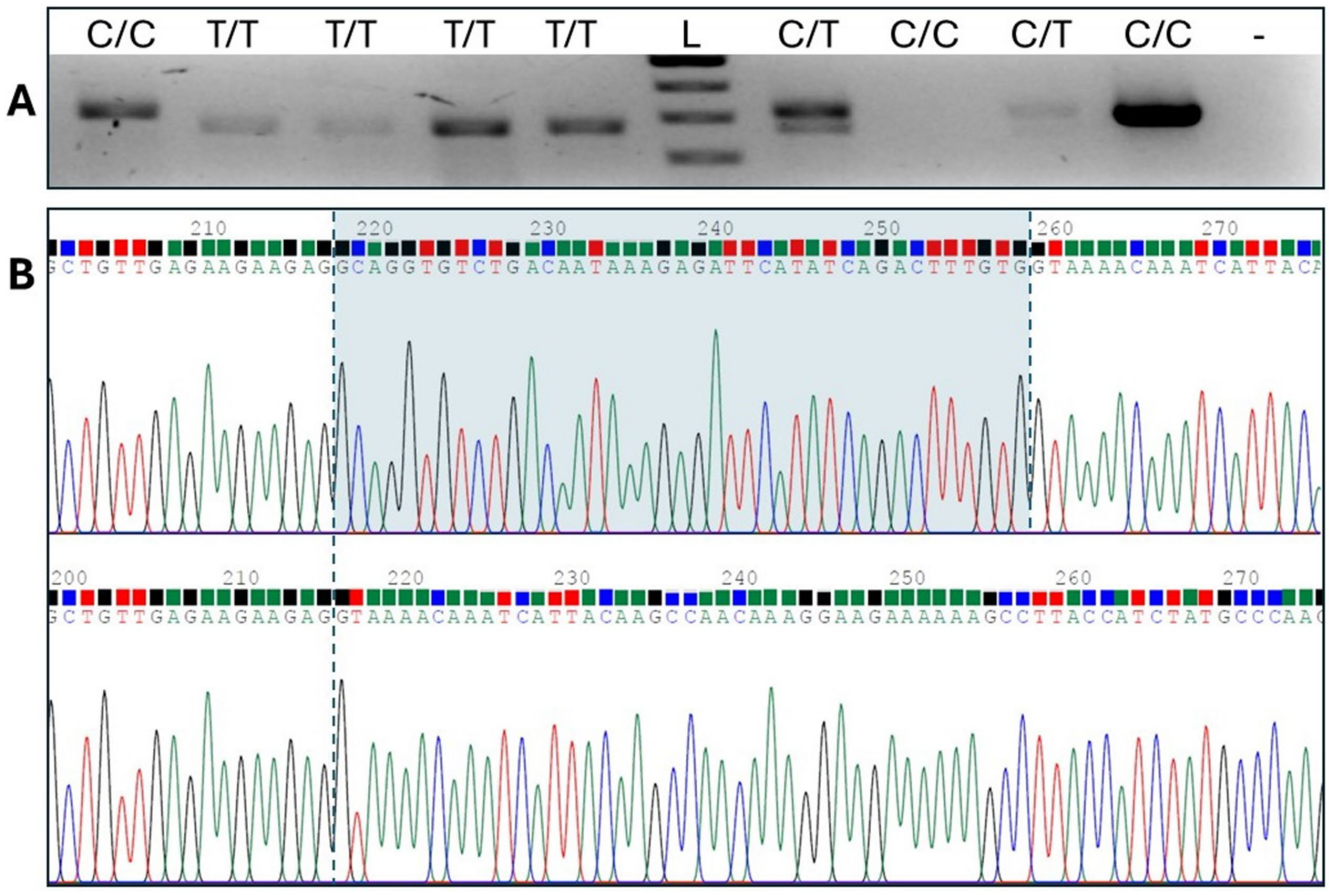
RT-PCR spanning exons 3,4, and 5. **(A)** Gel electrophoresis shows C/C homozygotes with an additional sequence compared to T/T homozygotes. Lanes 1, 3, and 4 represent the cDNA samples from the Boston Terriers, and lanes 2, 5, 7, 8, 9, and 10 represent the cDNA samples from the French Bulldogs. Lane L signifies a 100 bp ladder, and lane - represents the no-template control. **(B)** Sequence analysis of the large and small fragments identified an aberrant string of 41 bases in the *SLAMF1* transcript.

*SLAMF1* gene expression was assessed using RNA from canine blood. Exon-spanning RT-PCR yielded a PCR product larger than predicted for C/C homozygotes, suggesting that the wild-type splice site had indeed been disrupted and that an alternative, cryptic donor splice site downstream was being adopted. Sanger sequencing, which was used to compare the fragments of different lengths, revealed a 41 bp addition to exon 4 (Figure 4), resulting in a predicted aberrant run of 83 amino acids before termination (Supplementary file S2).

### Odds ratio, mode of inheritance, and dog 10 k data analysis

Odds ratios were calculated using the GWAS top SNPs and *SLAMF1* c799 + 2 T > 2 genotypes for a subset of cases and controls from the French Bulldog and Boxer breeds. The best mode of inheritance (MOI) models with odds ratios are shown in Table 1. A summary of the genotype data and all mode of inheritance determination calculations can be found in Supplementary file S4. Data from the Dog10k project were used to identify other breeds in which the *SLAMF1* c799 + 2 T > C variant was present (30). This variant was found in a total of 91 breeds and free-roaming dogs from Mexico, Azerbaijan, Liberia, Fiji, French Polynesia, Congo, Kenya, and China (Supplementary Table S4).

**Table 1:** Mode of inheritance and odds ratio analysis for the top SNPs and the putative *SLAMF1* variant.

Breed	Variant	Chr: Position (CF4)	MOI (best)	OR (95% CI)
FBD	BICF2G63068122	38:22420361 (rs24021480)	Additive	1.66 [1.46–1.88]
FBD	*SLAMF1* c799 + 2 T > C	38:22499957 (rs24034251)	Additive	1.89 [1.00–3.71]
Boxer	BICF2P422110	38:22263306 (rs24020434)	Additive	1.74 [1.44–2.1]
Boxer	SLAMF1 c799 + 2 T > C	38:22499957 (rs24034251)	Additive	2.75 [1.48–5.43]

## Discussion

In this study, a *SLAMF1* splice donor variant (*SLAMF1*:c799 + 2 T > C) associated with allergic dermatitis was identified through GWAS analysis, followed by whole-genome resequencing. The splice donor site involved is highly conserved across vertebrate species, suggesting that its disruption is likely to be deleterious. Furthermore, cDNA sequencing showed that the splice donor variant results in cryptic splicing, putting the transcript out of frame, which is predicted to result in a run of aberrant amino acids. This results in the replacement of the cytoplasmic tail of *SLAMF1*, including functional ITSM motifs that act as binding sites for cell signaling ligands (33). A predicted complete modification of the cytoplasmic tail would prevent SLAM-associated protein (SAP) binding and the inhibition of the SLAM-SAP-Fyn-SH3 ternary complex (34). When SAP binding to SLAMF1 is compromised, it could have significant consequences for immune responses, particularly those involving T cells and NK cells (35).

*SLAMF1*, or signaling lymphocytic activation molecule 1, is an immune system regulatory protein, also designated as CD150, expressed on the cell surface of T, B, NK, and dendritic cells (36). *SLAMF1* is part of a family of SLAM receptors and SLAM-associated protein (SAP) intracellular adaptors that play an active role in the immune system (34). It is well established that the measles virus entry is facilitated by SLAMF1 and CD46 as cellular receptors (37, 38). Interestingly, a potential link between measles and atopic dermatitis has been established, showing that human keratinocytes can be infected by the measles virus, modulating the expression of cytokines involved in allergic conditions such as atopic dermatitis (39). Furthermore, it has been demonstrated that vaccination against measles results in a protective effect against the development of atopic dermatitis. The canine distemper virus, a single-stranded RNA virus, is part of the same family as the measles virus. Given the high frequency of the *SLAMF1*:c799 + 2 T > C allele and the evidence that measles infection and vaccination provide a protective effect, it could be hypothesized that a defective SLAMF1 receptor provides some protection against canine distemper infection while increasing the risk of developing atopic dermatitis. SLAMF1 receptors act as self-ligands (40). Evidence that SLAM/SLAM interactions inhibit C40-induced production of inflammatory cytokines in monocyte-derived dendritic cells, a specialized immune system cell found in tissues including the skin, adds to the theory that deleterious variants in *SLAMF1* could reduce the regulation of inflammatory responses after a trigger event (41).

*SLAMF1* has been associated with several disease processes in humans, including rheumatoid arthritis (42), systemic lupus erythematosus (43), and diabetes (44, 45). These associations further establish *SLAMF1* involvement in autoimmune processes. Studies have identified increased odds of comorbidity for rheumatoid arthritis in patients with atopic dermatitis (46) and lupus in patients with atopic dermatitis (47). Although no direct link has been established between *SLAMF1* and atopic dermatitis, there is reasonable evidence suggesting that *SLAMF1* is involved in related disease processes. Pathways involving SLAM family members have already been investigated as potential therapeutic targets (48). For example, in a clinical pilot study, treatment with alefacept (a CD58-IgG1 fragment crystallizable (Fc) domain fusion protein) was found to reduce skin inflammation in cases of atopic eczema. Alefacept binds to CD2, a member of the CD2/SLAM gene family, with results suggesting reduced T cell activation after therapy (49). It has also been shown that the activation of SLAM by an mAb agonist in Th2 cells derived from the skin of patients with atopic dermatitis results in stable populations of IFN-gamma-producing cells that do not support IgE synthesis, potentially attenuating the allergic process. These results support the SLAM family as potential therapeutic targets for Th2 allergic disease (50).

The *SLAMF1*:c799 + 2 T > C variant, has an allele frequency of 0.082 in the Dog10k genome release (30) and is commonly found in popular dog breeds, such as the French Bulldog, Boxer, and Boston Terrier. It was found to increase the risk of allergic dermatitis by approximately 2-fold in our study, although additional studies are needed to fully understand the risk across all impacted breeds. It is already well established that atopic dermatitis is a disease with a complex etiology, and the presence of the variant in both case and control populations supports this. Both environmental factors and additional breed-specific genetic risk factors are likely to contribute to disease progression.

A clinical manifestation of canine atopic dermatitis may be complicated by secondary infections with yeast or bacteria (51). Failure to resolve these secondary infections may exacerbate the clinical signs of dermatitis. A Study on a *SLAMF1*−/− TCR knockout mice showed that *SLAMF1* is required for resistance to environmental fungal infections (52). There may be a possibility that *SLAMF1*:c799 + 2 T > C has a dual effect— increasing both the risk of an initial presentation of atopic dermatitis and the development of secondary yeast infections.

Atopic dermatitis is a common and significant welfare issue encountered in veterinary practice (53). Identifying a genetic risk factor may help improve our understanding of the disease process and potentially lead to more targeted therapeutics in the future. The identification of the *SLAMF1*:c799 + 2 T > C variant also presents an opportunity for breeders to breed dogs with a lower risk of developing atopic dermatitis. However, the frequency of the disease-associated variant is high and even potentially fixed in some breeds, as observed in the Dog10k data (30). It will be crucial to consider the maintenance of diversity as breeders seek to reduce the risk of atopic dermatitis in their breeding lines. It is also important that the association between *SLAMF1*:c799 + 2 T > C and CAD within a particular breed is confirmed before test results are used for selective breeding purposes, as CAD is a complex disease with modifiers likely altering the *SLAMF1*:c799 + 2 T > C-associated risk in each breed. However, given the discomfort the condition causes, genetic testing should not be discouraged, and breeders should be empowered with the tools and education they need to improve welfare.

In addition to the *SLAMF1* variant identified, significant associations were also established between atopic dermatitis and the dog leukocyte antigen (DLA) region. DLA involvement has been widely associated with autoimmune diseases in dogs, and the link between allergic dermatitis and the DLA region is a logical one, supported by a large-scale atopic dermatitis GWAS in humans (54). A genome-wide significant association was also identified on canine chromosome 7. While this association was not identified in the within-breed GWAS, it is a notable finding because it is located in a relatively gene-sparse region of the genome. The gene closest to the top SNPs on chromosome 7 is *FASLG* (Fas Ligand), which has previously been associated with several allergic disorders, including allergic rhinitis, psoriasis, asthma, hay fever, and eczema (55–57). Therefore, *FASLG* is an excellent positional and functional candidate gene in a relatively gene-sparse region of the dog genome, with only one other gene—SUN domain-containing ossification factor (SUCO)—located within 300 kb of the top SNPs on chromosome 7. However, a study using a higher-density array or using imputation would be needed to more finely map the chromosome 7-associated region.

In summary, this study identified a *SLAMF1*:c799 + 2 T > C splice donor variant associated with allergic dermatitis in domestic dogs. The study was made possible by a large clinical genetic dataset, highlighting the number of individuals needed to confidently uncover risk factors for highly polygenic disorders such as atopic dermatitis. In conclusion, this discovery represents a major risk factor for atopic dermatitis, as it is commonly diagnosed in primary veterinary practice.

## Data Availability

Original datasets are available in a publicly accessible repository: The original contributions presented in the study are publicly available. This data can be found here: https://doi.org/10.5061/dryad.np5hqc053 and European Nucleotide Archive (ENA) accession number PRJEB86959.

## Supplementary material

SUPPLEMENTARY FILE S1 All combined breed and single breed GWAS Manhattan Plots and QQ plots.

SUPPLEMENTARY FILE S2 Wild-type and variant *SLAMF1* transcripts and predicted amino acid sequence analysis.

SUPPLEMENTARY FILE S3 PCA for the combined single breeds GWAS demonstrating the relationship between cases and controls and clustering of the different breed groups.

SUPPLEMENTARY FILE S4 Top chr38 associated SNP and *SLAMF1* putative variant association analysis.

SUPPLEMENTARY TABLE S1 Breeds included in the combined single-breed GWAS.

SUPPLEMENTARY TABLE S2 Chromosome 38 regional genotype data for the 78 genome-sequenced dogs, indicating haplotypic cases.

SUPPLEMENTARY TABLE S3 Variant calls from whole genome sequence analysis post SNPeff and SNPsift analyses, sorted from low to high probability value.

SUPPLEMENTARY TABLE S4 Dog 10 k project SLAMF1 c799 + 2 T > C genotypes.
