# Correction: A splice donor variant in *SLAMF1* is associated with canine atopic dermatitis

**DOI:** 10.3389/fvets.2025.1668896

**Published:** 2025-08-19

**Authors:** Frontiers Production Office

**Affiliations:** Frontiers Media SA, Lausanne, Switzerland

**Keywords:** CAD, atopy, dermatitis, canine, allergy, SLAMF1, atopic dermatitis, allergic dermatitis

Supplementary material including Supplementary File 1, Supplementary File 2, Supplementary File 3, and Supplementary File 4, was omitted from the published article. The files have now been published.

The original version of this article has been updated.

