# Supplementary material for: A splice donor variant in SLAMF1 is associated with canine atopic dermatitis
Source: Front Vet Sci. 2025 Jun 19;12:1550617. doi: 10.3389/fvets.2025.1550617 (PMC12221898; doi:10.3389/fvets.2025.1550617)

# All GWAS Manhattan and QQ plots.

| Breed | Cases | Controls | Markers after QC | Genome Wide Significance threshold |
| --- | --- | --- | --- | --- |
| Balanced all pure breeds | 14,378 | 14,633 | 91,544 | 5.46E-07 |
| Beagle | 349 | 362 | 90,004 | 5.56E-07 |
| Boston Terrier | 478 | 531 | 88,793 | 5.63E-07 |
| Boxer | 447 | 515 | 84,538 | 5.91E-07 |
| Chihuahua | 304 | 363 | 91,269 | 5.48E-07 |
| Dachshund | 436 | 507 | 90,490 | 5.53E-07 |
| French Bull Dog | 2,138 | 645 | 84,541 | 5.91E-07 |
| German Shepherd Dog | 1,494 | 1,599 | 87,979 | 5.68E-07 |
| Golden Retriever | 1,056 | 1,057 | 84,914 | 5.89E-07 |
| Great Dane | 215 | 212 | 86,656 | 5.77E-07 |
| Japanese Shiba Inu | 239 | 284 | 80,686 | 6.20E-07 |
| Labrador Retriever | 1,668 | 1,064 | 89,363 | 5.60E-07 |
| Pembroke Welsh Corgi | 398 | 477 | 83,795 | 5.97E-07 |
| Poodle Medium/Standard | 461 | 498 | 90,239 | 5.54E-07 |
| Poodle Miniature/Toy | 385 | 464 | 91,030 | 5.49E-07 |
| Pug | 428 | 448 | 84,020 | 5.95E-07 |
| Schnauzer Miniature | 559 | 631 | 88,877 | 5.63E-07 |
| Shih Tzu | 797 | 929 | 89,006 | 5.62E-07 |
| Siberian Husky | 227 | 315 | 89,004 | 5.62E-07 |
| Yorkshire Terrier | 663 | 786 | 90,477 | 5.53E-07 |

All single breeds: 14,378 cases, 14,633 controls


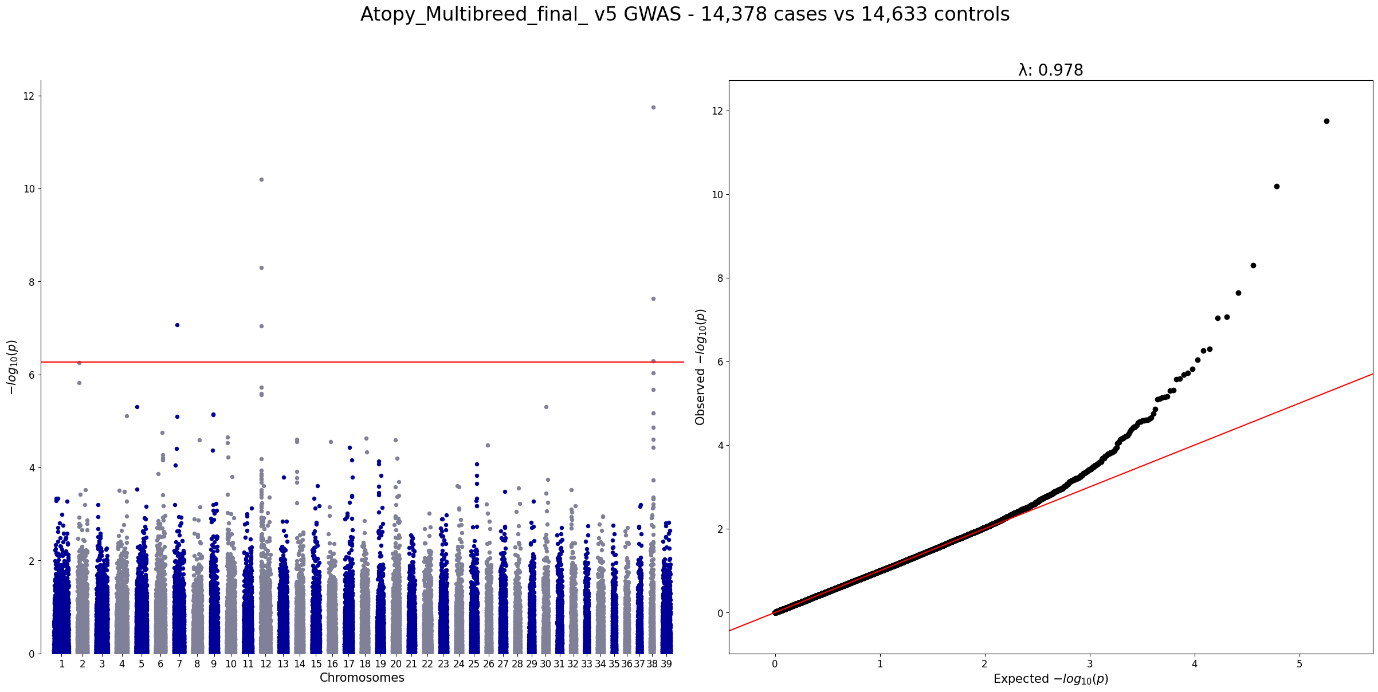


Beagle: 349 cases, 362 controls


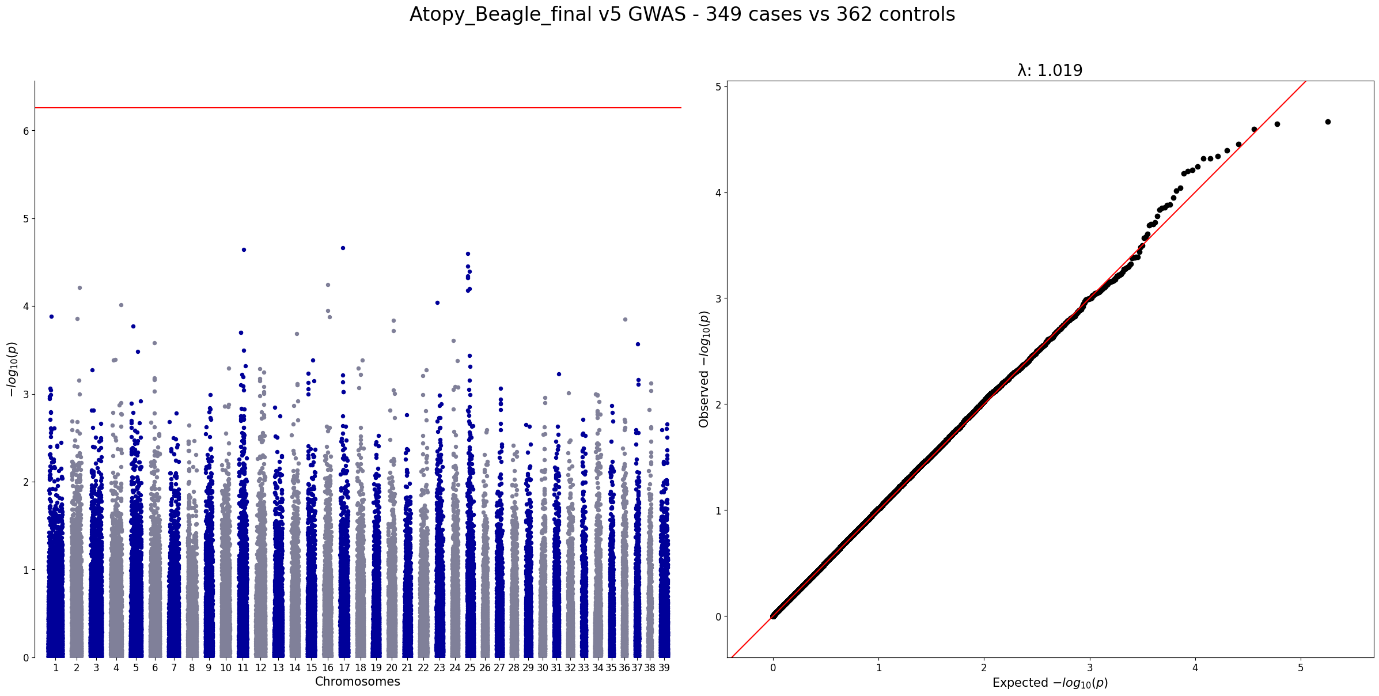


Boston Terrier: 478 cases, 531 controls


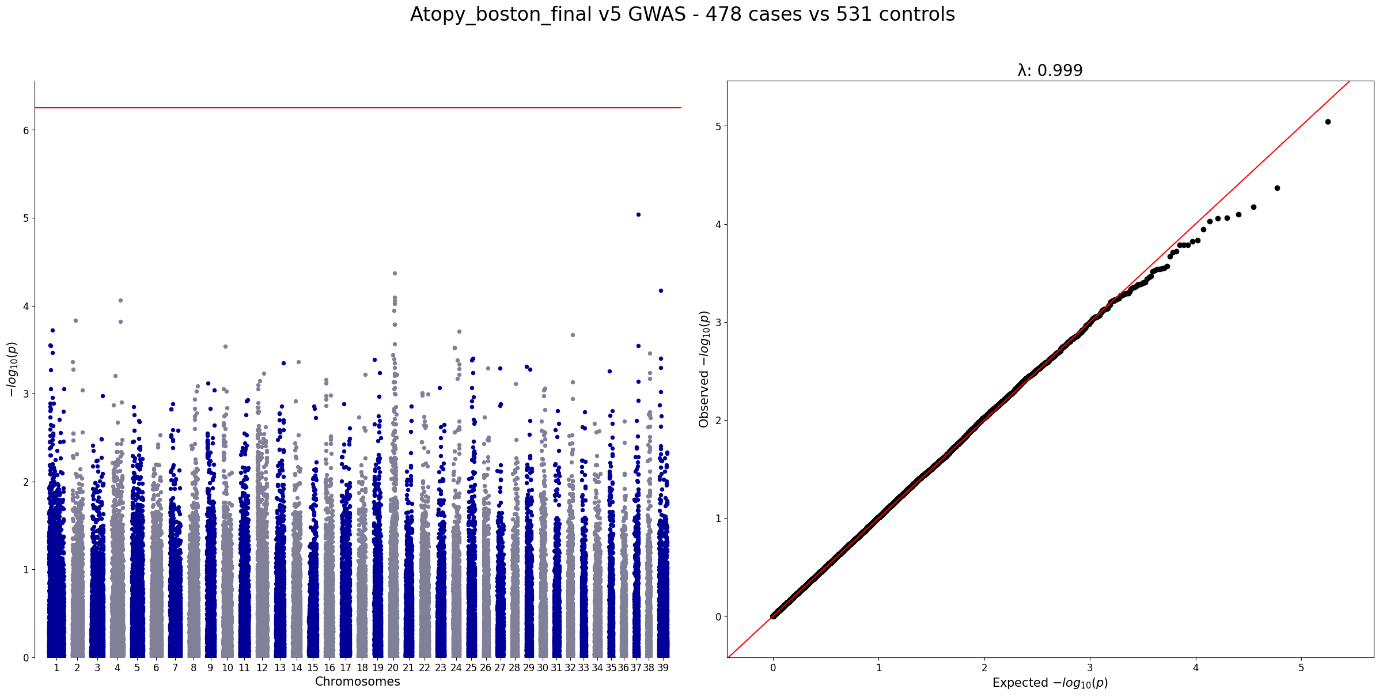


Boxer: 447 cases, 515 controls


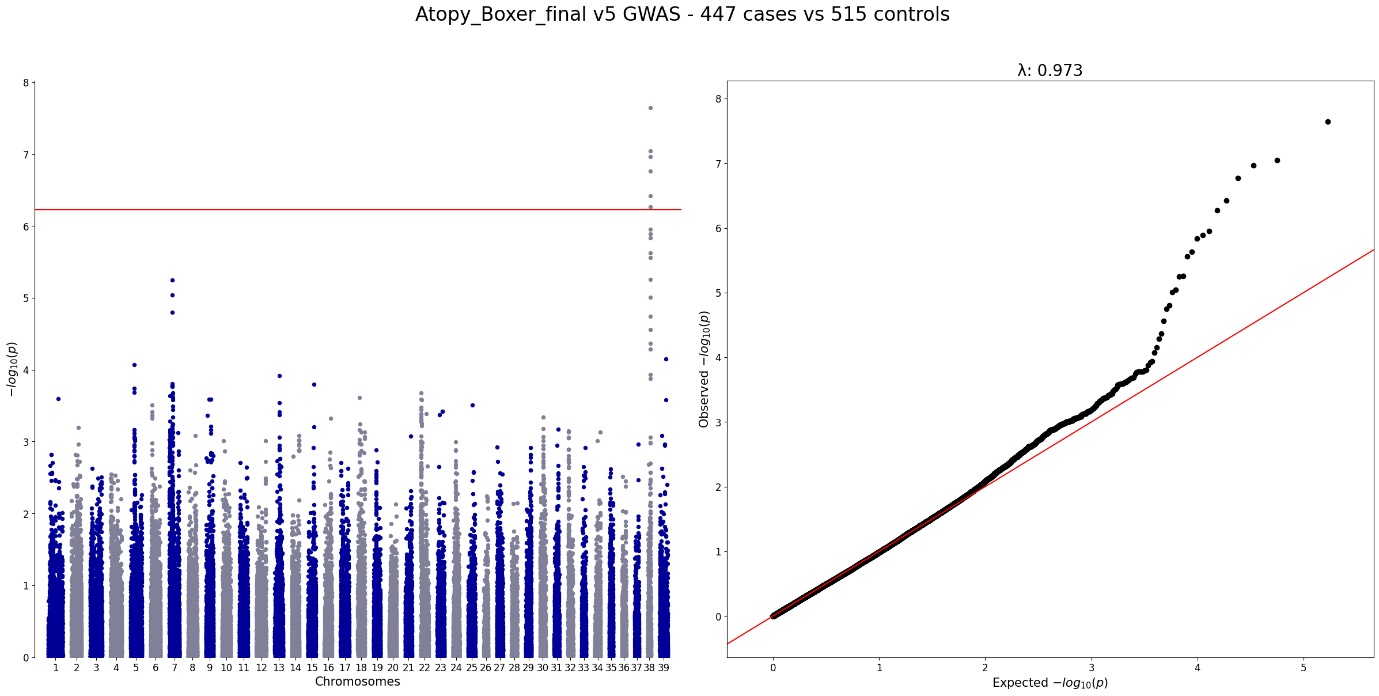


Chihuahua: 304 cases, 363 controls


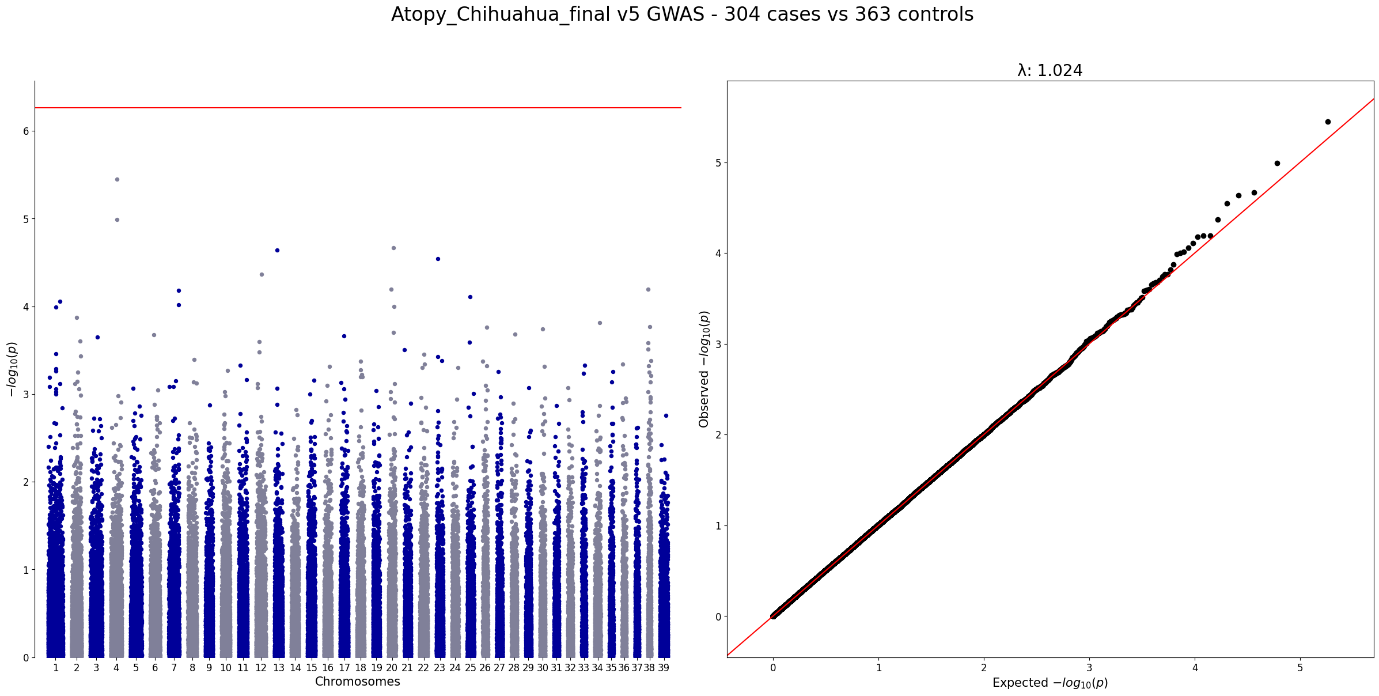


Dachshund: 436 cases, 507 controls


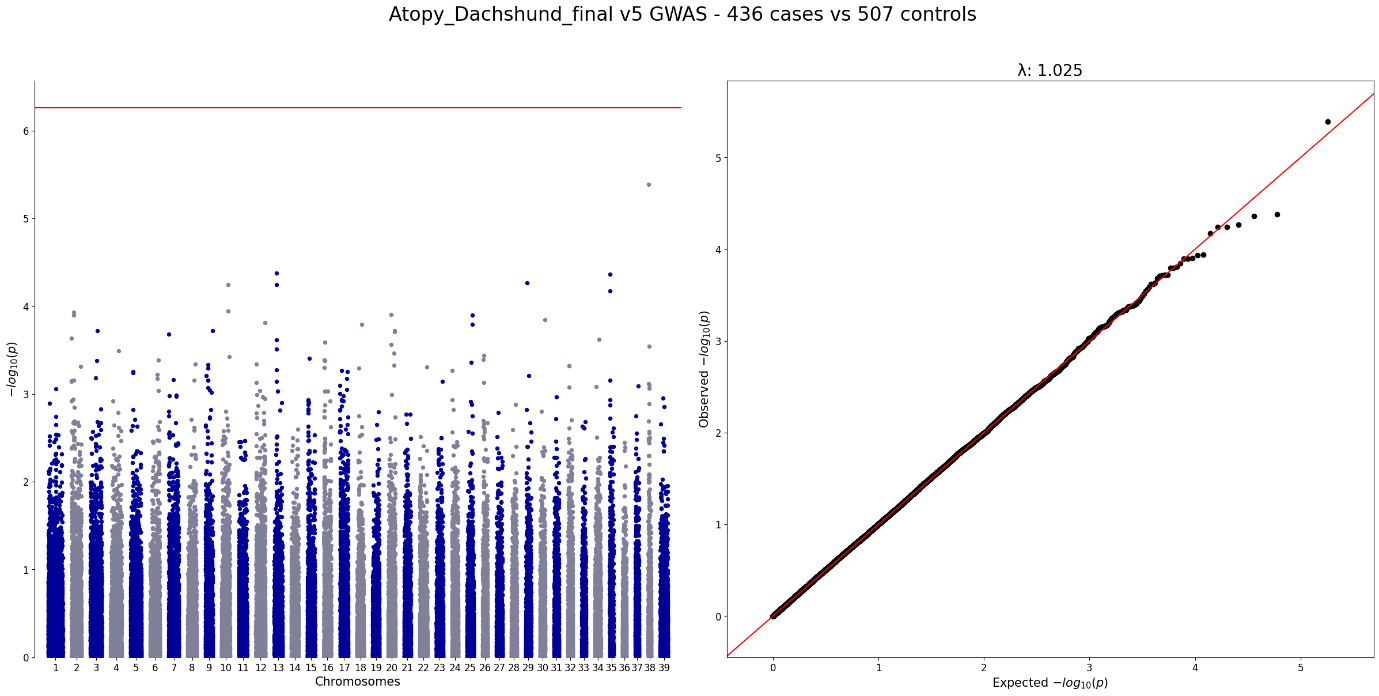


French Bull Dog: 2,138 cases, 645 controls


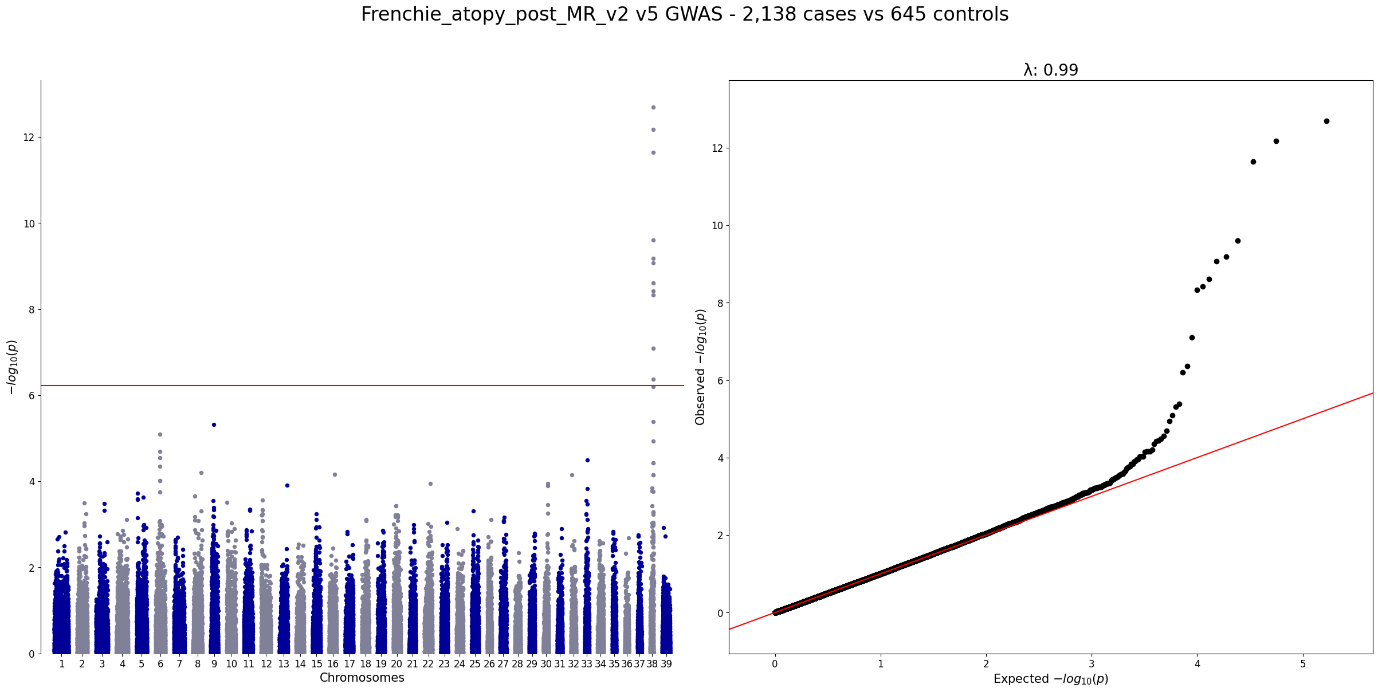


Golder Retriever: 1,056 cases, 1,057 controls


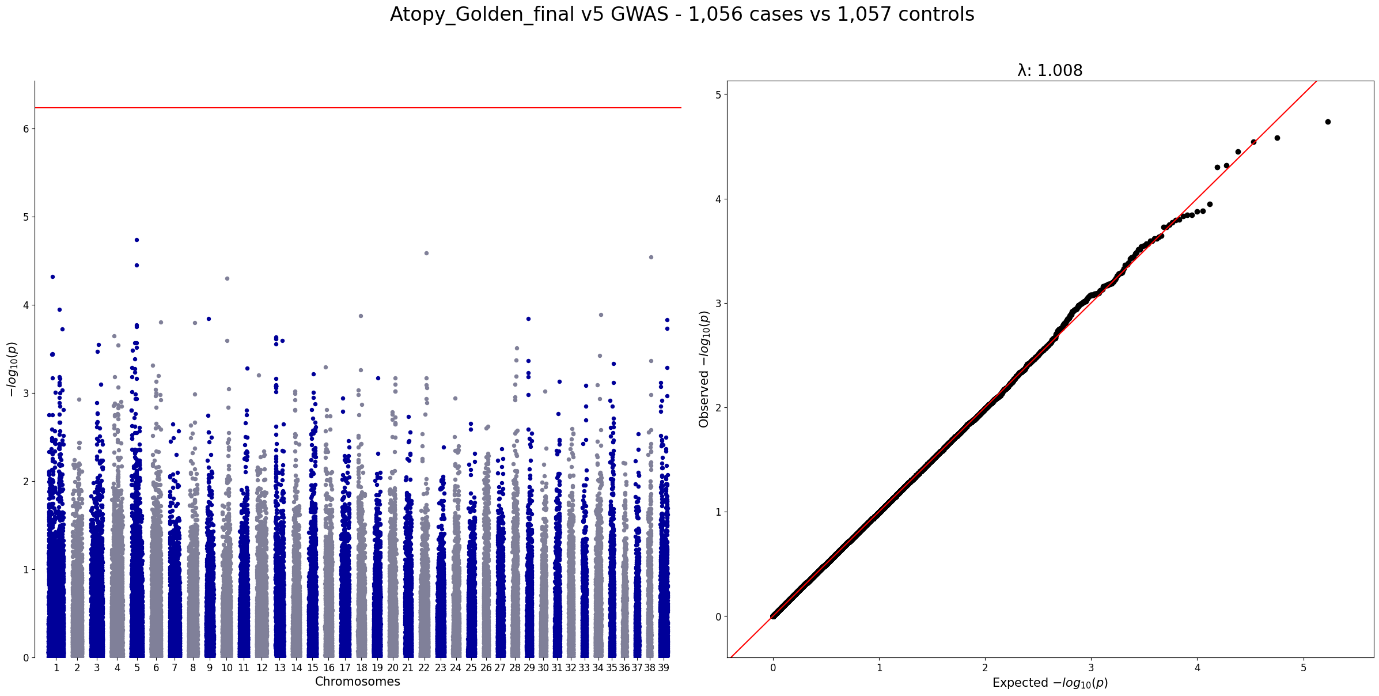


German Shepherd Dog: 1,494 cases, 1,599 controls


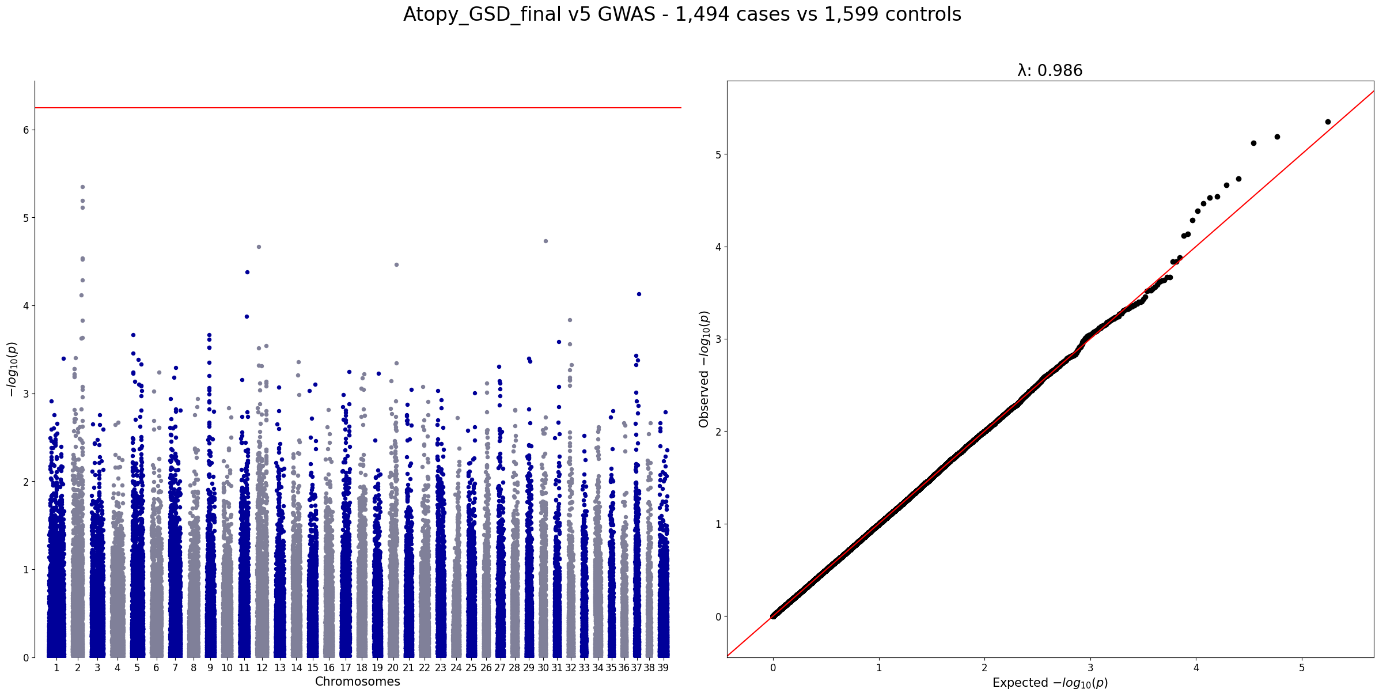


Great Dane: 215 cases, 212 controls


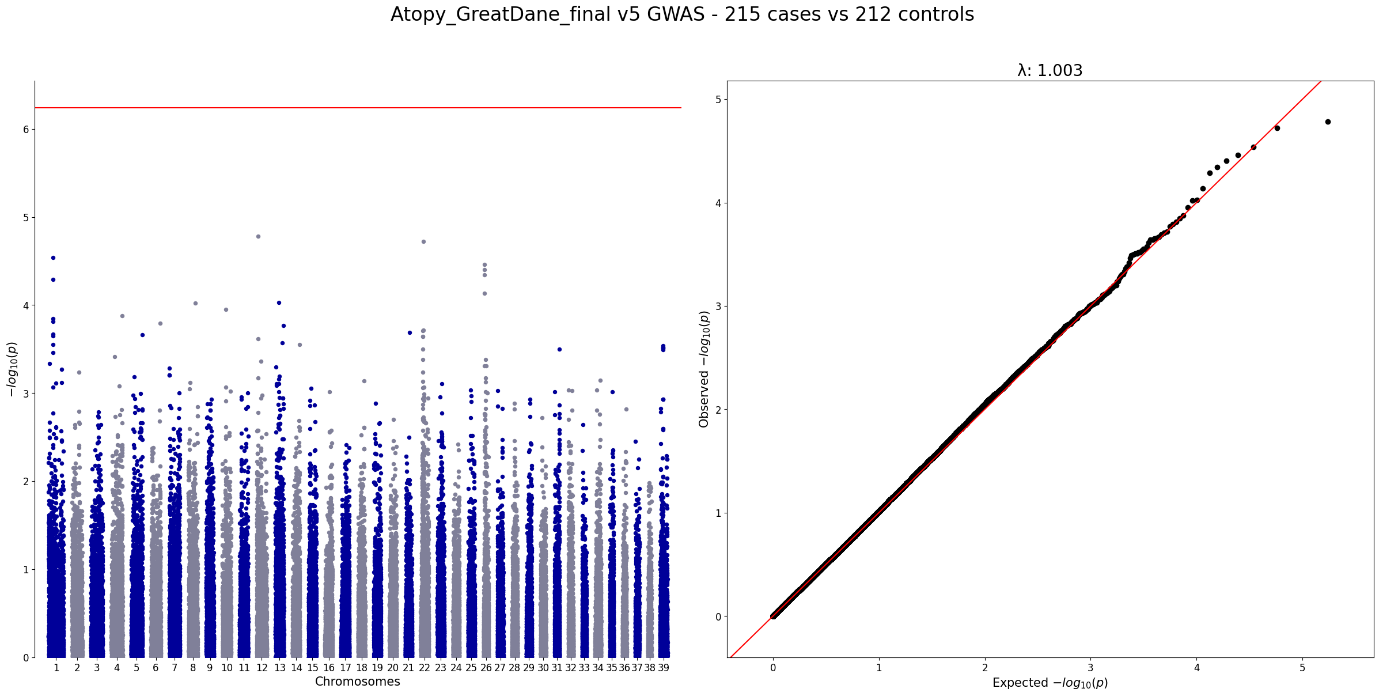


Japanese Shiba Inu: 239 cases, 284 controls


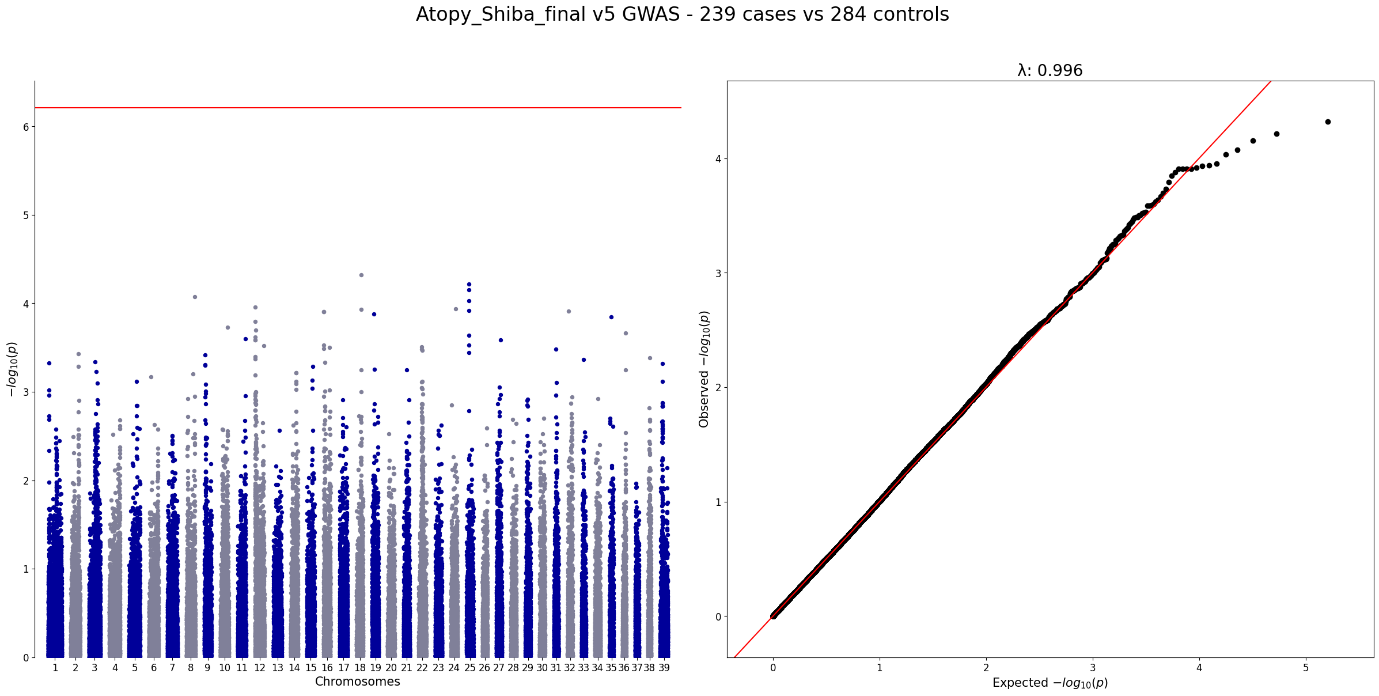


Labrador Retriever: 1,668 cases, 1,064 controls


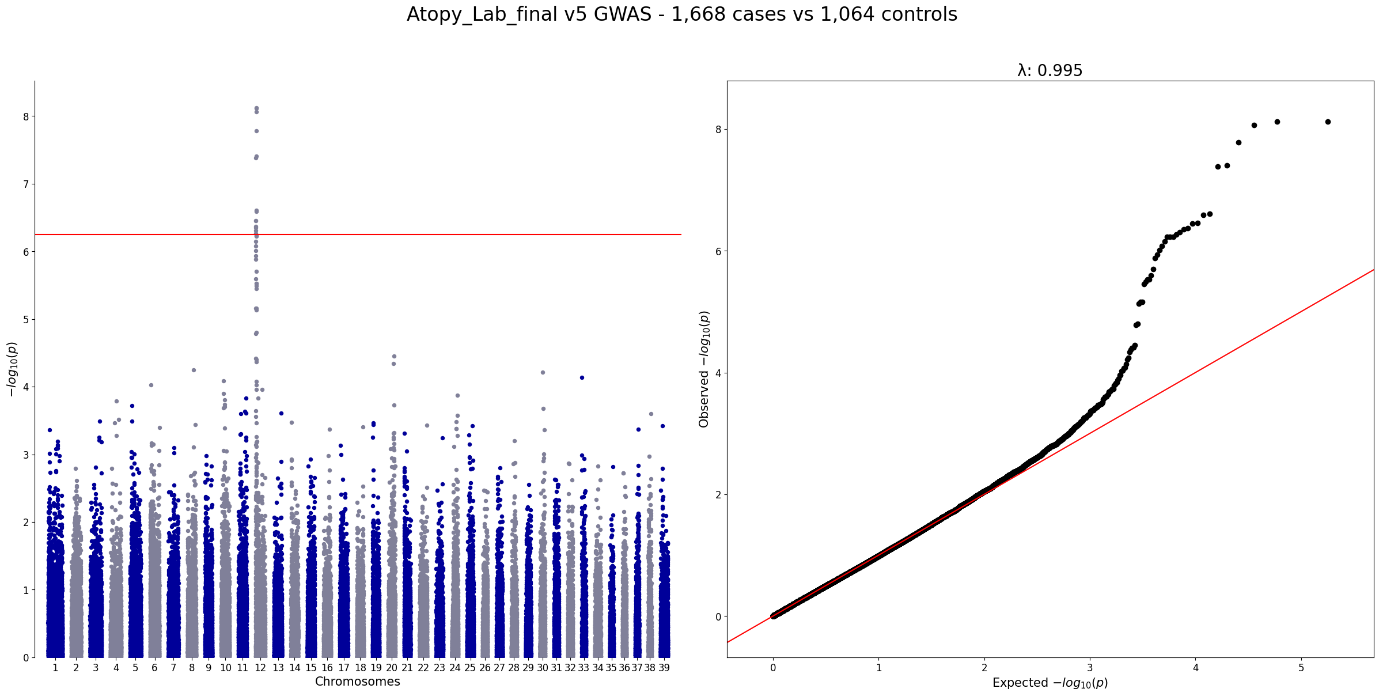


Miniature Poodle: 385 cases, 464 controls


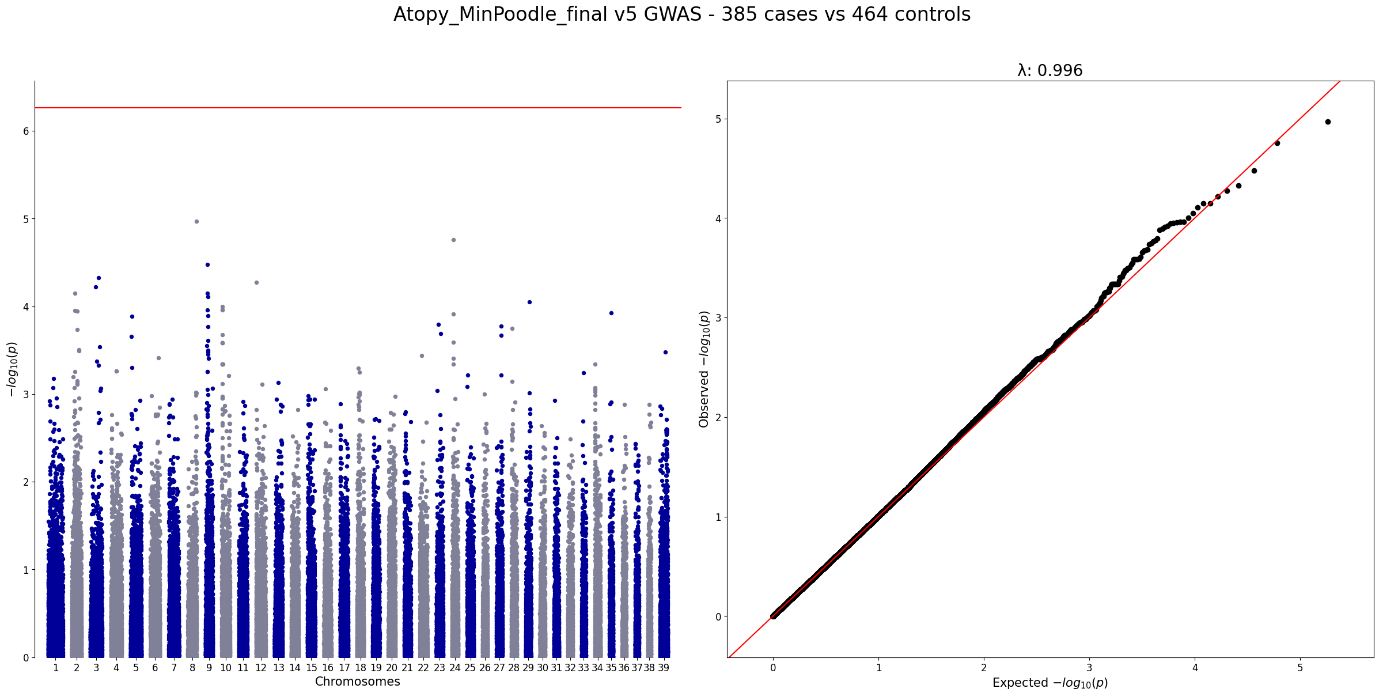


Miniature Schnauzer: 559 cases, 631 controls


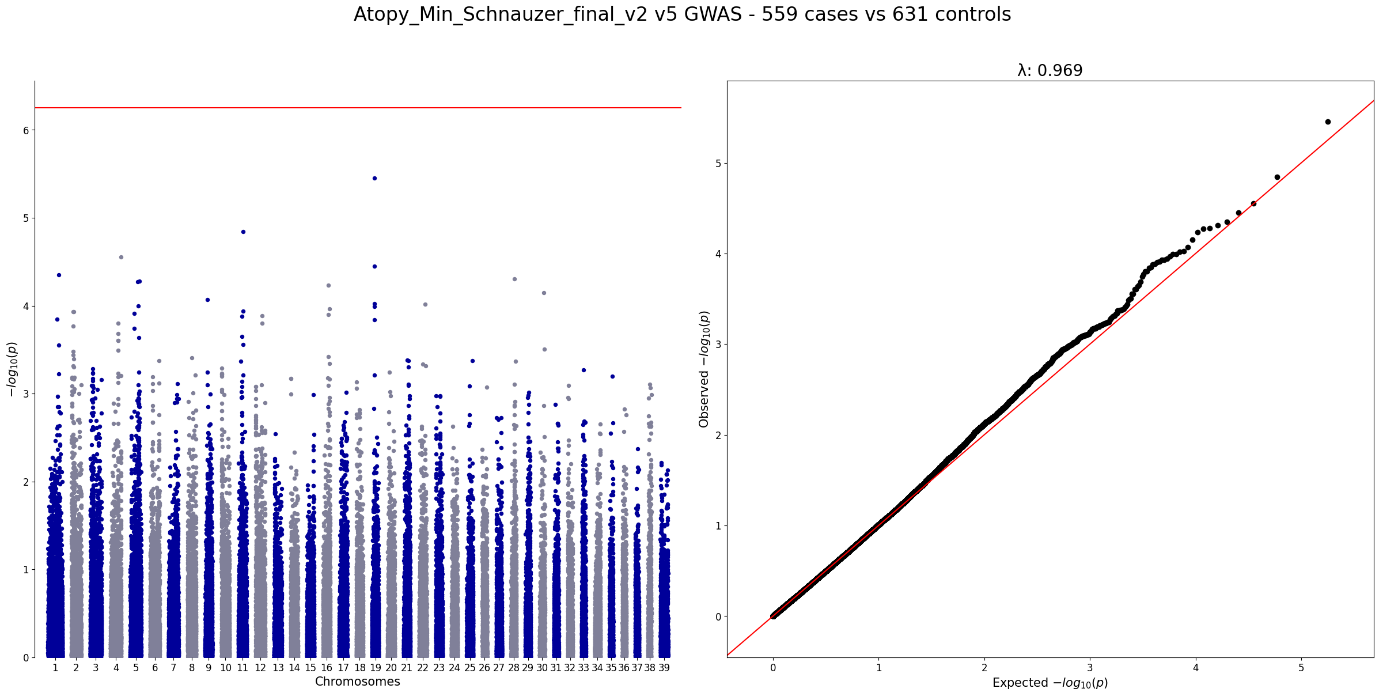


Pembroke Welsh Corgi: 398 cases, 477 controls


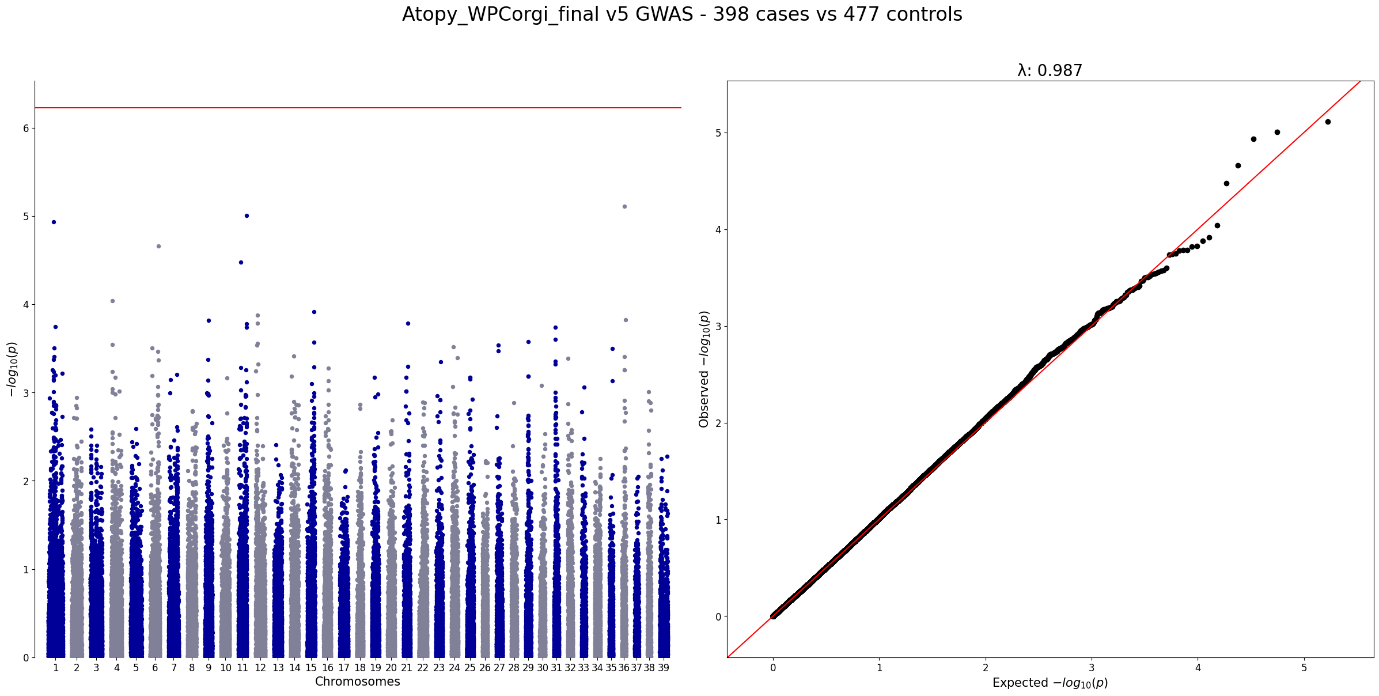


Pug: 428 cases, 448 controls


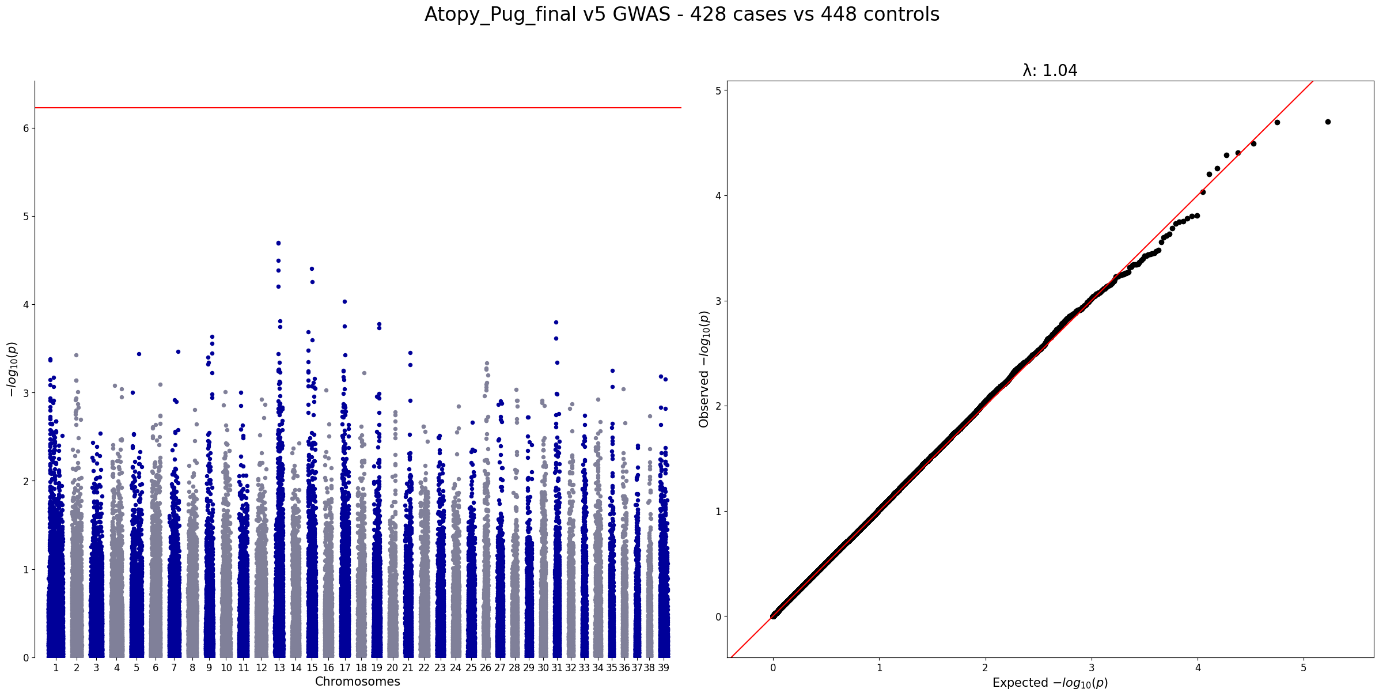


ShihTzu: 797 cases, 929 controls


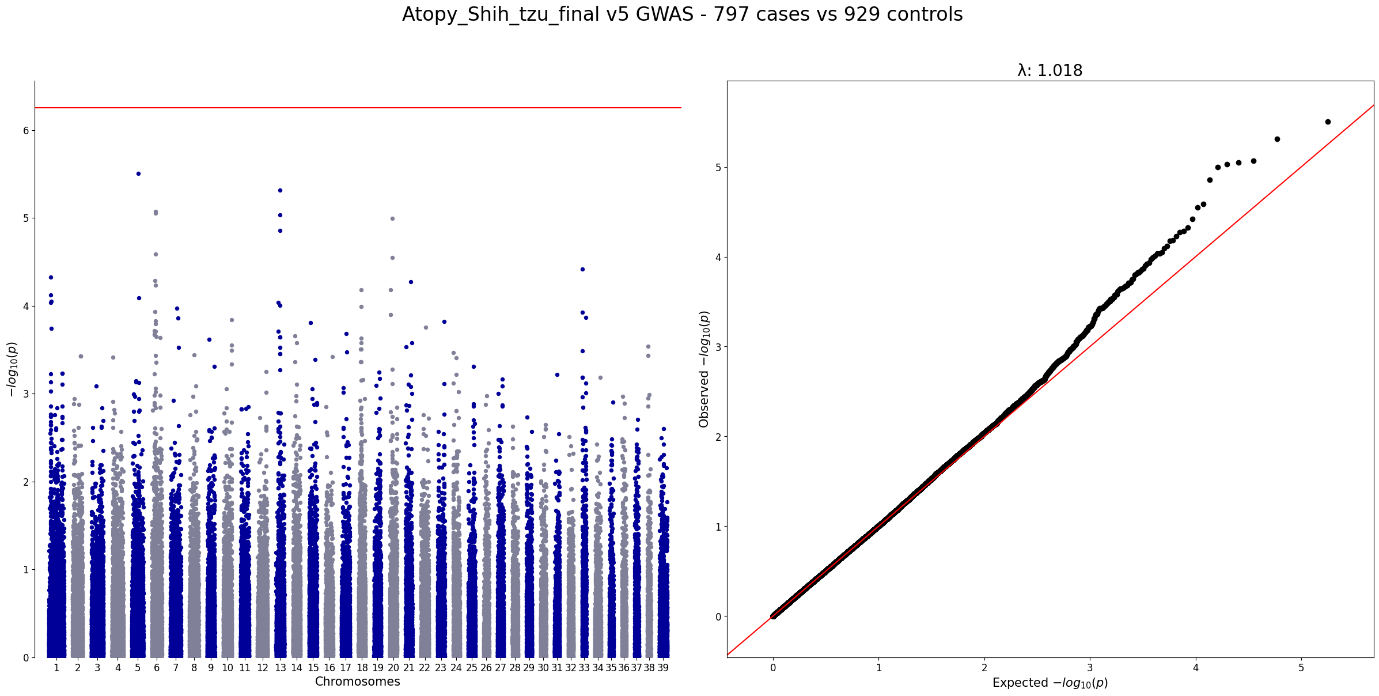


Siberian Husky: 227 cases, 315 controls


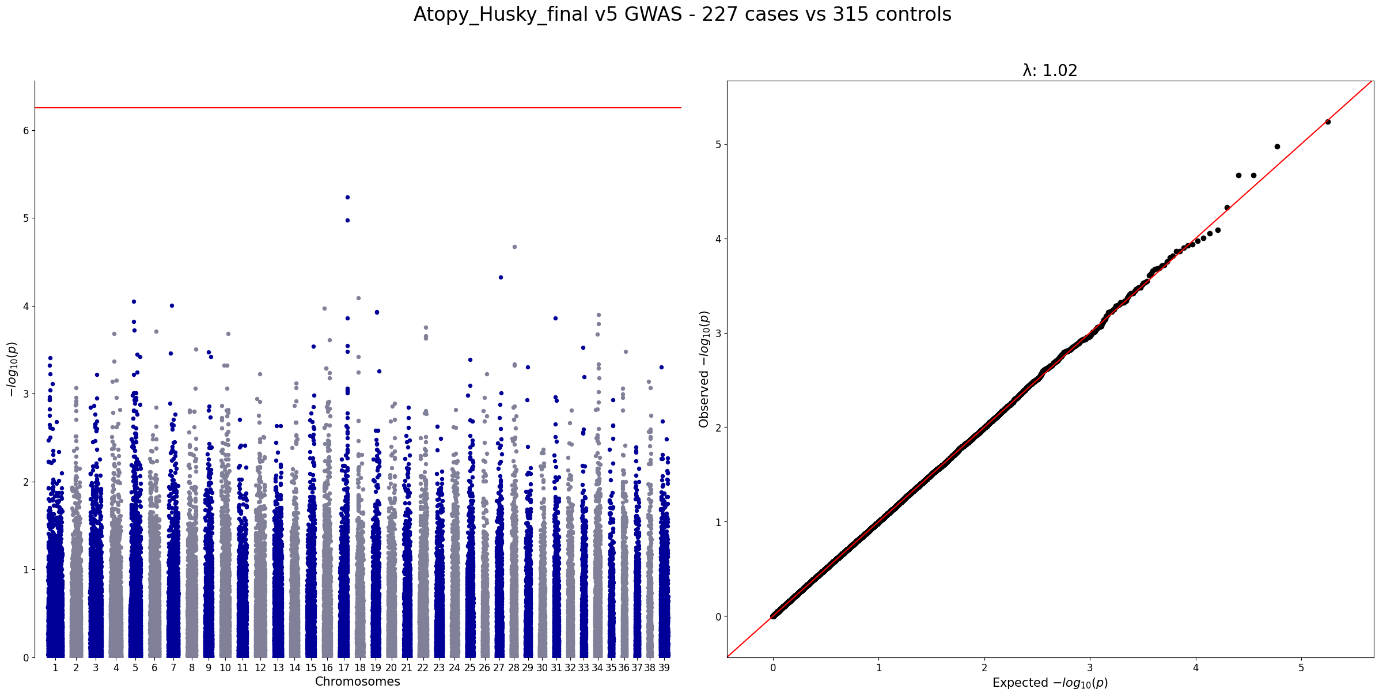


Standard and Medium Poodle: 461 cases, 498 controls


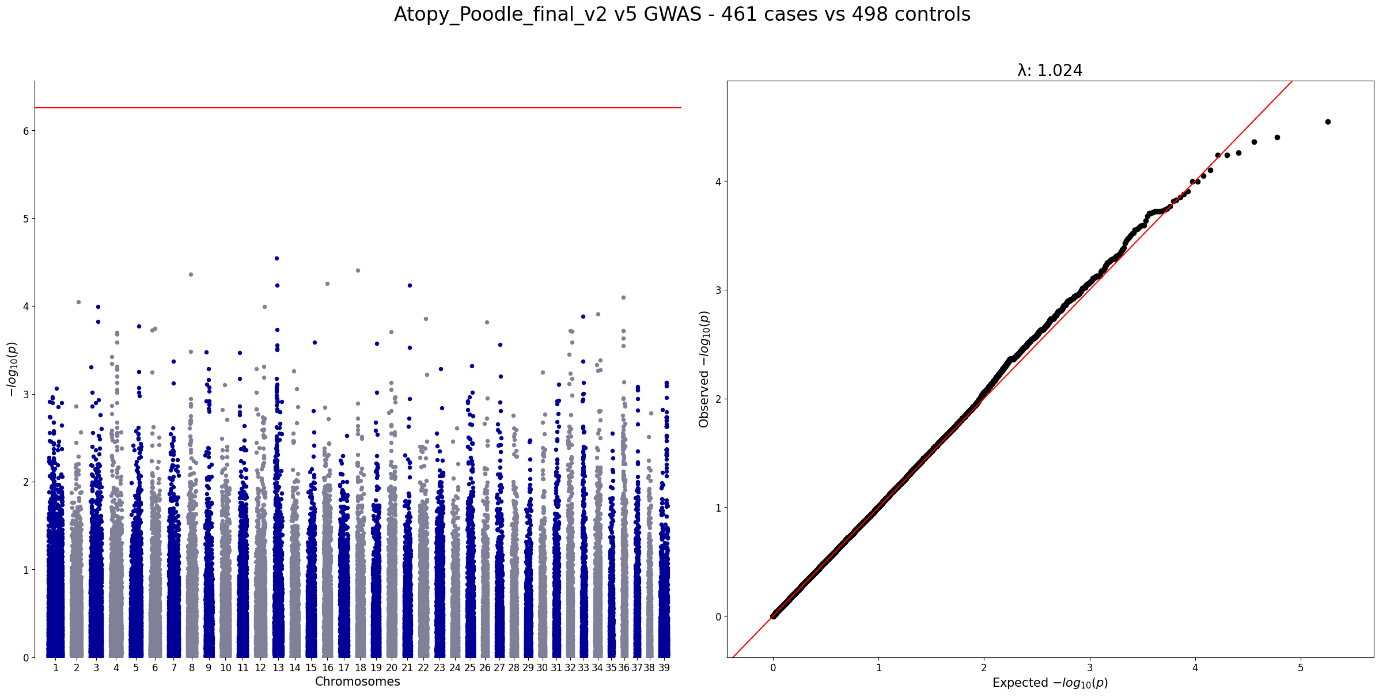


Yorkshire terrier: 663 cases, 786 controls


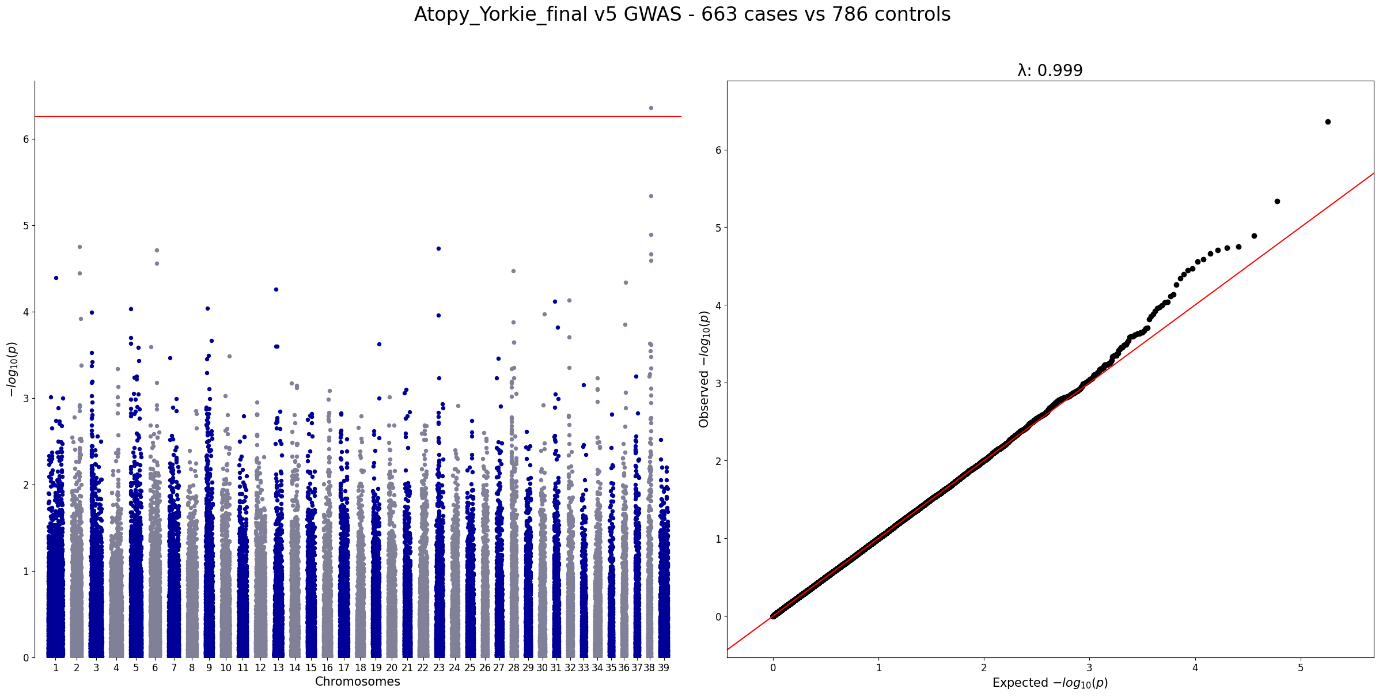

Supplement: SUPPLEMENTARY FILE S1 — All combined breed and single breed GWAS Manhattan Plots and QQ plots. [file Supplementary_file_1.zip › Supplementary File 1-4/Supplementary File 1-4/Supplementary File S1.docx]
