# Supplementary material for: A splice donor variant in SLAMF1 is associated with canine atopic dermatitis
Source: Front Vet Sci. 2025 Jun 19;12:1550617. doi: 10.3389/fvets.2025.1550617 (PMC12221898; doi:10.3389/fvets.2025.1550617)
Supplement: SUPPLEMENTARY FILE S1 — All combined breed and single breed GWAS Manhattan Plots and QQ plots. [file Supplementary_file_1.zip › Supplementary File 1-4/Supplementary File 1-4/Supplementary File S2.docx]

**RNA samples for SLAMF1 transcript investigations**

| Internal ID | Gel Lane | Conc. (ng/µl) | RIN | Breed | Genotype |
| --- | --- | --- | --- | --- | --- |
| 799 | 1 | 83 | 7.6 | Boston Terrier | CC |
| 800 | 2 | 19.01 | 5.4 | French Bull Dog | TT |
| 801 | 3 | 98.37 | 5.5 | Boston Terrier | TT |
| 802 | 4 | 74.19 | 7.6 | Boston Terrier | TT |
| 803 | 5 | 39.27 | 7.7 | French Bull Dog | TT |
| 804 | 7 | 51.05 | 5.3 | French Bull Dog | CT |
| 805 | 8 | 66.98 | 6.6 | French Bull Dog | CC |
| 806 | 9 | 21.17 | 6.5 | French Bull Dog | CT |
| 807 | 10 | 33.4 | 8.6 | French Bull Dog | CC |

**Canine Wild Type and Variant Transcript Comparison**

**ATG** - START

**TGA** – STOP

**TGA** – VARIANT STOP

GCAGGTGTCTGACAATAAAGAGATTCATATCAGACTTTGTG **-** ABERRANT SEQUENCE

**Wild Type Canine *SLAMF1* transcript sequence**

ATGGATTCCAGGGGCTTCCTCTCCCTGCGCTGCCTGCTGGTCCTCGCCCTGGCGTCCAAGCTGAGCTGTGGGACAGGTGAGAGCTTGATGAATTGCCCAGAAGTCCCTGGGAAGTTGGGAAGCAGTTTGCAGCTGTCCTTGGCATCCGAAGGGATAAGCAAGAGGATGAACAAGAGCATCCACATCCTTGTCACAAGGGCGGAATCACCGGGAAACAGTATCAAGAAGAAAATAGTGTCTCTGGATCTGCCAGAAGGGGGGTCTCCACGCTACCTGGAAAATGGCTATAAATTTCATCTGGAAAACCTGACCCTGAGGATCCTGGAAAGCAGGAGGGAAAATGAAGGCTGGTACTTCATGACCCTGGAGGAGAACTTTTCAGTTCAACACTTTTGTCTGCAGCTGAAGCTCTATGAGCAGGTCTCCACTCCAGAAATTAAGGTGTTGAACTGGACCCAGGAGAATGGGAACTGCAGCATGATGCTGGCCTGCGAAGTGGAAAAGGGGGACAATGTGGTTTACAGCTGGAGTGAGAAACTGGGGATTGACCCACTGATCCCAGCCAACAGTTCTCACCTCTTGCACCTCAGCCTCGGCCCTCAGCATGTCAACAACGTCTATGTCTGCACCGTGAGCAACCCCGTCAGCAACCGCTCATGGTCCTTCAACCCATGGTCCAAGTGCAGGCCAGAGTCTTCGGTGCCAAGACAATGGAGACTGTATGCTGGGCTCTTCTTAGGGGGTATCGTTGGTGTCATCTTGATTTTCGAAGTGGTATTACTGCTGTTGAGAAGAAGAGGTAAAACAAATCATTACAAGCCAACAAAGGAAGAAAAAAGCCTTACCATCTATGCCCAAGTCCAGAAATCAGGTTCTACTCAGAAGAAACCTGATCCCTTGCCAGCTGAGGACCCCTGCACCACCATTTATGTTGCTGCCACAGAACCTGTCCCAGAACCTGCCCCAGAACCTGTCCAGGAACCACATTCTATCACGGTCTATGCCAGCGTGACGTTCCCAGAGAGCTGA

**Variant Canine *SLAMF1* transcript sequence**

ATGGATTCCAGGGGCTTCCTCTCCCTGCGCTGCCTGCTGGTCCTCGCCCTGGCGTCCAAGCTGAGCTGTGGGACAGGTGAGAGCTTGATGAATTGCCCAGAAGTCCCTGGGAAGTTGGGAAGCAGTTTGCAGCTGTCCTTGGCATCCGAAGGGATAAGCAAGAGGATGAACAAGAGCATCCACATCCTTGTCACAAGGGCGGAATCACCGGGAAACAGTATCAAGAAGAAAATAGTGTCTCTGGATCTGCCAGAAGGGGGGTCTCCACGCTACCTGGAAAATGGCTATAAATTTCATCTGGAAAACCTGACCCTGAGGATCCTGGAAAGCAGGAGGGAAAATGAAGGCTGGTACTTCATGACCCTGGAGGAGAACTTTTCAGTTCAACACTTTTGTCTGCAGCTGAAGCTCTATGAGCAGGTCTCCACTCCAGAAATTAAGGTGTTGAACTGGACCCAGGAGAATGGGAACTGCAGCATGATGCTGGCCTGCGAAGTGGAAAAGGGGGACAATGTGGTTTACAGCTGGAGTGAGAAACTGGGGATTGACCCACTGATCCCAGCCAACAGTTCTCACCTCTTGCACCTCAGCCTCGGCCCTCAGCATGTCAACAACGTCTATGTCTGCACCGTGAGCAACCCCGTCAGCAACCGCTCATGGTCCTTCAACCCATGGTCCAAGTGCAGGCCAGAGTCTTCGGTGCCAAGACAATGGAGACTGTATGCTGGGCTCTTCTTAGGGGGTATCGTTGGTGTCATCTTGATTTTCGAAGTGGTATTACTGCTGTTGAGAAGAAGAGGCAGGTGTCTGACAATAAAGAGATTCATATCAGACTTTGTGGTAAAACAAATCATTACAAGCCAACAAAGGAAGAAAAAAGCCTTACCATCTATGCCCAAGTCCAGAAATCAGGTTCTACTCAGAAGAAACCTGATCCCTTGCCAGCTGAGGACCCCTGCACCACCATTTATGTTGCTGCCACAGAACCTGTCCCAGAACCTGCCCCAGAACCTGTCCAGGAACCACATTCTATCACGGTCTATGCCAGCGTGACGTTCCCAGAGAGCTGA

**Canine Wild Type and Variant Protein Comparison**

**Wild Type Canine *SLAMF1* translated amino acid sequence**

**MDSRGFLSLRCLLVLALASKLSCGTGESLMNCPEVPGKLGSSLQLSLASEGISKRMNKSIHILVTRAESPGNSIKKKIVSLDLPEGGSPRYLENGYKFHLENLTLRILESRRENEGWYFMTLEENFSVQHFCLQLKLYEQVSTPEIKVLNWTQENGNCSMMLACEVEKGDNVVYSWSEKLGIDPLIPANSSHLLHLSLGPQHVNNVYVCTVSNPVSNRSWSFNPWSKCRPESSVPRQWRLYAGLFLGGIVGVILIFEVVLLLLRRRGKTNHYKPTKEEKSLTIYAQVQKSGSTQKKPDPLPAEDPCTTIYVAATEPVPEPAPEPVQEPHSITVYASVTFPES***

**Variant Canine *SLAMF1* translated amino acid sequence**

**MDSRGFLSLRCLLVLALASKLSCGTGESLMNCPEVPGKLGSSLQLSLASEGISKRMNKSIHILVTRAESPGNSIKKKIVSLDLPEGGSPRYLENGYKFHLENLTLRILESRRENEGWYFMTLEENFSVQHFCLQLKLYEQVSTPEIKVLNWTQENGNCSMMLACEVEKGDNVVYSWSEKLGIDPLIPANSSHLLHLSLGPQHVNNVYVCTVSNPVSNRSWSFNPWSKCRPESSVPRQWRLYAGLFLGGIVGVILIFEVVLLLLRRRGRCLTIKRFISDFVVKQIITSQQRKKKALPSMPKSRNQVLLRRNLIPCQLRTPAPPFMLLPQNLSQNLPQNLSRNHILSRSMPA***

**Human and Canine Protein Comparison**

Source: <https://www.uniprot.org/uniprotkb/Q13291/feature-viewer>

**Motif:**

TIYAQV – ITSM1

YVAATE – SH2-binding

TVYASV – ITSM2

**Human SLAMF1**

MDPKGLLSLTFVLFLSLAFGASYGTGGRMMNCPKILRQLGSKVLLPLTYERINKSMNKSIHIVVTMAKSLENSVENKIVSLDPSEAGPPRYLGDRYKFYLENLTLGIRESRKEDEGWYLMTLEKNVSVQRFCLQLRLYEQVSTPEIKVLNKTQENGTCTLILGCTVEKGDHVAYSWSEKAGTHPLNPANSSHLLSLTLGPQHADNIYICTVSNPISNNSQTFSPWPGCRTDPSETKPWAVYAGLLGGVIMILIMVVILQLRRRGKTNHYQTTVEKKSLTIYAQVQKPGPLQKKLDSFPAQDPCTTIYVAATEPVPESVQETNSITVYASVTLPES

**Canine SLAMF1**

MDSRGFLSLRCLLVLALASKLSCGTGESLMNCPEVPGKLGSSLQLSLASEGISKRMNKSIHILVTRAESPGNSIKKKIVSLDLPEGGSPRYLENGYKFHLENLTLRILESRRENEGWYFMTLEENFSVQHFCLQLKLYEQVSTPEIKVLNWTQENGNCSMMLACEVEKGDNVVYSWSEKLGIDPLIPANSSHLLHLSLGPQHVNNVYVCTVSNPVSNRSWSFNPWSKCRPESSVPRQWRLYAGLFLGGIVGVILIFEVVLLLLRRRGKTNHYKPTKEEKSLTIYAQVQKSGSTQKKPDPLPAEDPCTTIYVAATEPVPEPAPEPVQEPHSITVYASVTFPES

**Pairwise Alignment**

<https://www.ebi.ac.uk/jdispatcher/psa/emboss_needle/>

########################################

# Program: needle

# Rundate: Thu 19 Dec 2024 12:11:06

# Commandline: needle

# -auto

# -stdout

# -asequence emboss_needle-I20241219-121103-0196-6984772-p1m.asequence

# -bsequence emboss_needle-I20241219-121103-0196-6984772-p1m.bsequence

# -datafile EBLOSUM62

# -gapopen 10.0

# -gapextend 0.5

# -endopen 10.0

# -endextend 0.5

# -aformat3 pair

# -sprotein1

# -sprotein2

# Align_format: pair

# Report_file: stdout

########################################

#=======================================

#

# Aligned_sequences: 2

# 1: EMBOSS_001

# 2: EMBOSS_001

# Matrix: EBLOSUM62

# Gap_penalty: 10.0

# Extend_penalty: 0.5

#

# Length: 342

# Identity: 222/342 (64.9%)

# Similarity: 266/342 (77.8%)

# Gaps: 7/342 ( 2.0%)

# Score: 1124.0

#

#

#=======================================

EMBOSS_001 1 MDPKGLLSLTFVLFLSLAFGASYGTGGRMMNCPKILRQLGSKVLLPLTYE 50

||.:|.|||..:|.|:||...|.|||..:||||::..:|||.:.|.|..|

EMBOSS_001 1 MDSRGFLSLRCLLVLALASKLSCGTGESLMNCPEVPGKLGSSLQLSLASE 50

EMBOSS_001 51 RINKSMNKSIHIVVTMAKSLENSVENKIVSLDPSEAGPPRYLGDRYKFYL 100

.|:|.|||||||:||.|:|..||::.||||||..|.|.||||.:.|||:|

EMBOSS_001 51 GISKRMNKSIHILVTRAESPGNSIKKKIVSLDLPEGGSPRYLENGYKFHL 100

EMBOSS_001 101 ENLTLGIRESRKEDEGWYLMTLEKNVSVQRFCLQLRLYEQVSTPEIKVLN 150

|||||.|.|||:|:||||.||||:|.|||.|||||:||||||||||||||

EMBOSS_001 101 ENLTLRILESRRENEGWYFMTLEENFSVQHFCLQLKLYEQVSTPEIKVLN 150

EMBOSS_001 151 KTQENGTCTLILGCTVEKGDHVAYSWSEKAGTHPLNPANSSHLLSLTLGP 200

.|||||.|:::|.|.|||||:|.||||||.|..||.||||||||.|:|||

EMBOSS_001 151 WTQENGNCSMMLACEVEKGDNVVYSWSEKLGIDPLIPANSSHLLHLSLGP 200

EMBOSS_001 201 QHADNIYICTVSNPISNNSQTFSPWPGCRTDPSETKPWAVYAGL-LGGVI 249

||.:|:|:||||||:||.|.:|:||..||.:.|..:.|.:|||| |||::

EMBOSS_001 201 QHVNNVYVCTVSNPVSNRSWSFNPWSKCRPESSVPRQWRLYAGLFLGGIV 250

EMBOSS_001 250 MILIM--VVILQLRRRGKTNHYQTTVEKKSLTIYAQVQKPGPLQKKLDSF 297

.:::: ||:|.||||||||||:.|.|:|||||||||||.|..|||.|..

EMBOSS_001 251 GVILIFEVVLLLLRRRGKTNHYKPTKEEKSLTIYAQVQKSGSTQKKPDPL 300

EMBOSS_001 298 PAQDPCTTIYVAATEPV----PESVQETNSITVYASVTLPES 335

||:|||||||||||||| ||.|||.:|||||||||.|||

EMBOSS_001 301 PAEDPCTTIYVAATEPVPEPAPEPVQEPHSITVYASVTFPES 342
