# Supplementary material for: A splice donor variant in SLAMF1 is associated with canine atopic dermatitis
Source: Front Vet Sci. 2025 Jun 19;12:1550617. doi: 10.3389/fvets.2025.1550617 (PMC12221898; doi:10.3389/fvets.2025.1550617)

PCA showing cases (yellow) and controls (dark blue) for the combined single breed GWAS approach


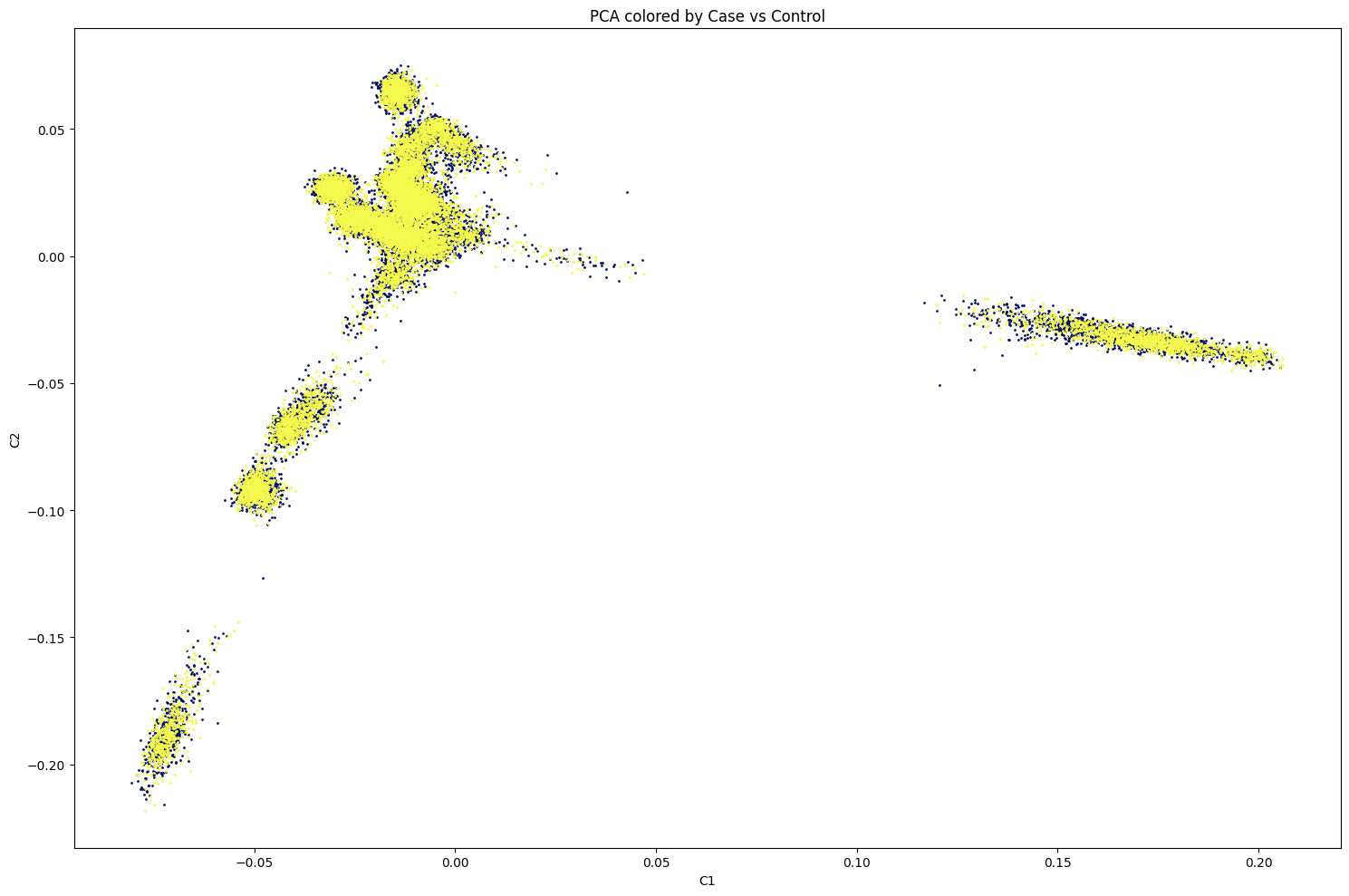


PCA colored by breed, demonstrating distinct subclustering.


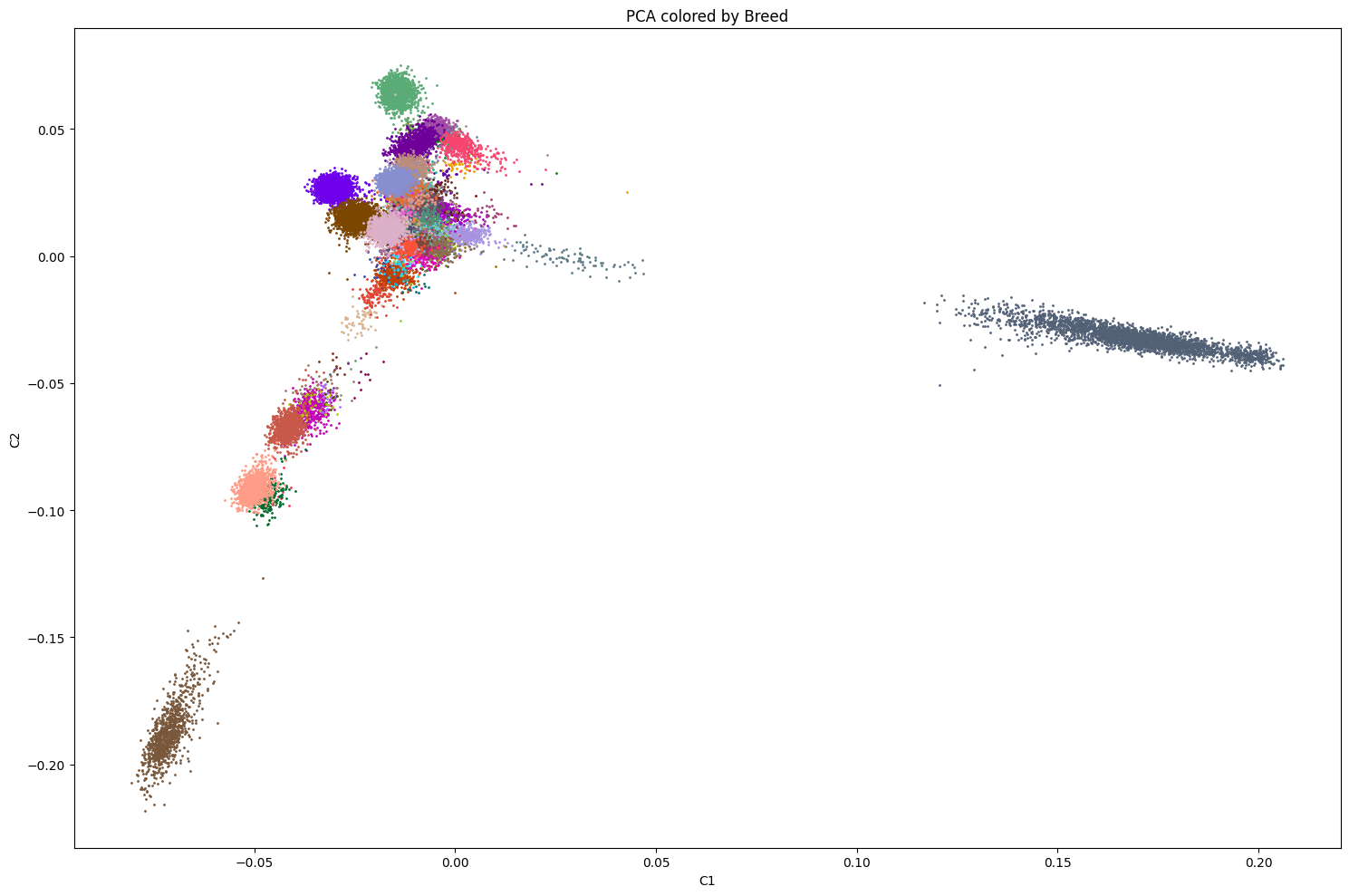


Key for PCA colored by breed.


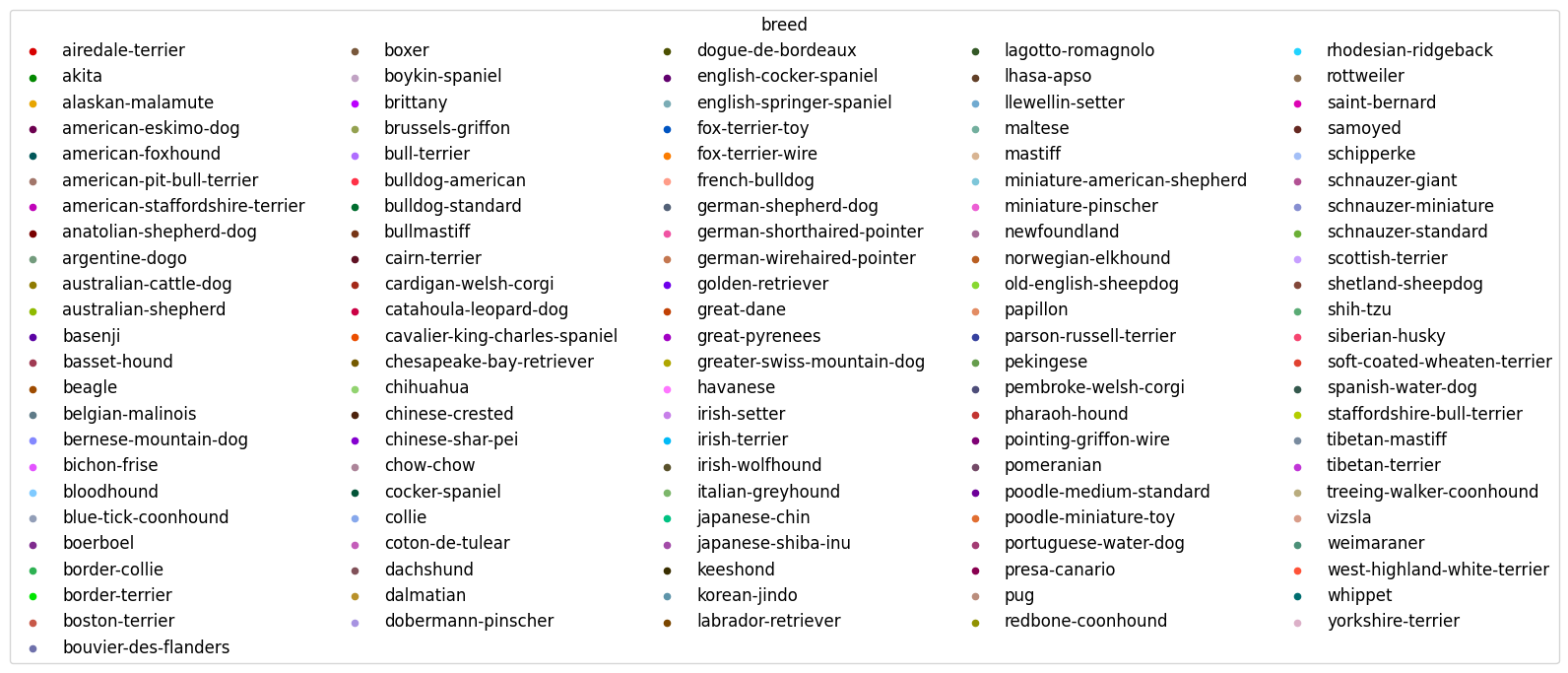

Supplement: SUPPLEMENTARY FILE S1 — All combined breed and single breed GWAS Manhattan Plots and QQ plots. [file Supplementary_file_1.zip › Supplementary File 1-4/Supplementary File 1-4/Supplementary File S3.docx]
