# Supplementary material for: A splice donor variant in SLAMF1 is associated with canine atopic dermatitis
Source: Front Vet Sci. 2025 Jun 19;12:1550617. doi: 10.3389/fvets.2025.1550617 (PMC12221898; doi:10.3389/fvets.2025.1550617)
Supplement: SUPPLEMENTARY FILE S1 — All combined breed and single breed GWAS Manhattan Plots and QQ plots. [file Supplementary_file_1.zip › Supplementary File 1-4/Supplementary File 1-4/Supplementary File S4.docx]

# TOP SNP ANALYSIS

**French Bull Dog Top SNP:**

|  | 38_21592552 (BICF2G63068122) Genotype | | |  |
| --- | --- | --- | --- | --- |
|  | 0 | 1 | 2 | Grand Total |
| Controls | 182 | 313 | 151 | 646 |
| Cases | 918 | 944 | 277 | 2139 |
| Grand Total | 1100 | 1257 | 428 | 2785 |

**MOI results:**

|  | P-value | OR [95% CI] |
| --- | --- | --- |
| Additive | 1.777e-15 | 1.66 [1.46-1.88] |
| Dominant | 5.994e-10 | 2.05 [1.64-2.56] |
| Recessive | 8.665e-12 | 1.92 [1.59-2.33] |

**Boxer Top SNP:**

|  | 38_21434822 (BICF2P422110) Genotype | | |  |
| --- | --- | --- | --- | --- |
|  | 0 | 1 | 2 | Grand Total |
| Controls | 101 | 234 | 181 | 516 |
| Cases | 39 | 182 | 226 | 447 |
| Grand Total | 140 | 416 | 407 | 963 |

**MOI results:**

|  | P-value | OR [95% CI] |
| --- | --- | --- |
| Additive | 4.542e-09 | 1.74 [1.44-2.1] |
| Dominant | 1.22e-06 | 2.55 [1.73-3.81] |
| Recessive | 1.199e-06 | 1.89 [1.46-2.45] |

# *SLAMF1* GENOTYPE ANALYSIS

A random subset of cases and controls were genotyped for the *SLAMF1* candidate variant.

**C is the disease-associated allele**

**French Bull Dog *SLAMF1* Genotype data**

|  | Cases | Controls |
| --- | --- | --- |
| C:C | 24 | 6 |
| C:T | 17 | 9 |
| T:T | 9 | 8 |
| All | 50 | 23 |

**MOI results:**

|  | P-value | OR [95% CI] |
| --- | --- | --- |
| Additive | 0.04974 | 1.89 [1-3.71] |
| Dominant | 0.123 | 2.43 [0.78-7.55 |
| Recessive | 0.082 | 2.62 [0.92-8.26] |

**Boxer *SLAMF1* Genotype data**

| Boxers | Cases | Controls |
| --- | --- | --- |
| C:C | 22 | 5 |
| C:T | 22 | 17 |
| T:T | 8 | 14 |
| All | 52 | 36 |

**MOI results:**

|  | P-value | OR [95% CI] |
| --- | --- | --- |
| Additive | 0.00105 | 2.75 [1.48-5.43] |
| Dominant | 0.01272 | 3.5 [1.3-9.98] |
| Recessive | 0.00662 | 4.55 [1.62-14.99] |
